# Neurostimulation devices to treat Alzheimer’s disease

**DOI:** 10.37349/en.2025.100674

**Published:** 2025-02-25

**Authors:** Felipe P. Perez, Brett Walker, Jorge Morisaki, Haitham Kanakri, Maher Rizkalla

**Affiliations:** 1 Department of Medicine, Division of General Internal Medicine and Geriatrics, Indiana University School of Medicine, Indianapolis, IN 46202, USA; 2 Department of Bioengineering, University of Illinois at Chicago, Chicago, IL 60607, USA; 3 Department of Electrical and Computer Engineering, Purdue University, Indianapolis, IN 46202, USA

**Keywords:** Alzheimer’s disease, treatment, electromagnetic fields stimulation, devices, preclinical, clinical, review

## Abstract

The use of neurostimulation devices for the treatment of Alzheimer’s disease (AD) is a growing field. In this review, we examine the mechanism of action and therapeutic indications of these neurostimulation devices in the AD process. Rapid advancements in neurostimulation technologies are providing non-pharmacological relief to patients affected by AD pathology. Neurostimulation therapies include electrical stimulation that targets the circuitry-level connection in important brain areas such as the hippocampus to induce therapeutic neuromodulation of dysfunctional neural circuitry and electromagnetic field (EMF) stimulation that targets anti-amyloid molecular pathways to promote the degradation of beta-amyloid (Aβ). These devices target specific or diffuse cortical and subcortical brain areas to modulate neuronal activity at the electrophysiological or molecular pathway level, providing therapeutic effects for AD. This review attempts to determine the most effective and safe neurostimulation device for AD and provides an overview of potential and current clinical indications. Several EMF devices have shown a beneficial or harmful effect in cell cultures and animal models but not in AD human studies. These contradictory results may be related to the stimulation parameters of these devices, such as frequency, penetration depth, power deposition measured by specific absorption rate, time of exposure, type of cell, and tissue dielectric properties. Based on this, determining the optimal stimulation parameters for EMF devices in AD and understanding their mechanism of action is essential to promote their clinical application, our review suggests that repeated EMF stimulation (REMFS) is the most appropriate device for human AD treatments. Before its clinical application, it is necessary to consider the complicated and interconnected genetic and epigenetic effects of REMFS-biological system interaction. This will move forward the urgently needed therapy of EMF in human AD.

## Introduction

As of 2022, an estimated 6.5 million Americans lived with Alzheimer’s disease (AD), and as the population ages, this number is expected to rise to 14 million by 2060. AD costs the nation $321 billion in 2022, and unless we develop an effective treatment, costs will continue to escalate, reaching $1 trillion in 2050 [1]. The standard of care for AD treatment includes cholinesterase inhibitors, memantine hydrochloride (*N*-methyl-*D*-aspartate receptor antagonist), and monoclonal antibodies (mAbs). However, these methods are unable to lower the toxic Aβ levels without causing brain swelling or microhemorrhages [2, 3], unable to stop AD progression [4], unable to reach multiple targets [5, 6], unable to easily cross the blood-brain-barrier (BBB) [7], and unable to link the aging process to AD pathology [8].

AD is described by beta-amyloid (Aβ) plaques and tangles of neurofibrillary tau proteins in the brain [9]. The amyloid hypothesis states that increases in Aβ levels cause the disease [9], specifically, the binding of Aβ oligomers to multiple cellular receptors is the probable cause of neuronal toxicity [10]. This induces mitochondrial dysfunction and oxidative stress in AD neurons [11]. A human study with 13C6-leucine demonstrated that the pathological factor in AD was a deficit in the clearance rather than the overproduction of Aβ from the central nervous system (CNS) to the periphery [12, 13]. This deficiency of plaque clearance progresses into neuronal degeneration, causing memory impairment and cognitive decline. Therefore, any therapy that has anti-amyloid properties that lower Aβ levels would likely prevent neurodegeneration [14] and cognitive impairment if it does not cause brain inflammation or bleeds. Preclinical studies showed that lowering Aβ levels by repeated electromagnetic field stimulation (REMFS) stops and reverses AD. Even though mAbs lower Aβ levels, they have not stopped AD progression due to serious side effects and the fact that they target extracellular Aβ [2, 3], instead of enhancing intracellular proteostasis or autophagy.

Numerous EMF devices have recently investigated the effects of exposure to EMFs on the underlying pathomolecular pathways of AD [15], leading to innovative therapeutic strategies into the mechanisms through which EMF modulates AD-related symptoms. Depending on the type of system, it can be classified as electric current (direct or alternating), magnetic stimulation (causes induction of electric field to depolarized neurons), and REMFS, which is based on the frequency used. EMFs can be classified in extremely low frequency (ELF) (3–30 Hz), super LF (SLF) (30–300 Hz), ultra LF (ULF) 300 Hz–3 kHz, very LF (VLF) 3–30 kHz, LF 30–300 kHz, medium frequency (MF) 3 kHz–3 MHz, high frequency (HF) 3–300 MHz, very HF (VHF) 30–300 MHz, ultra HF (UHF) 300 MHz–3 GHz, super HF (SHF) 3–30 GHz, extremely HF 30–300 GHz, and terahertz radiation 0.3–3 THz [16–18]. Electromagnetic pulses (EMPs) vs. continuous waves are another type of EMF and can be distinguished by their unique properties. Pulse EMF can affect alpha activity [16] and impair cognitive function [17, 18], whereas continuous EMF does not affect alpha activity or impair cognition. This review focuses on continuous wave REMFS. Depending on the amplitude, time of exposure, intensity, tissue characteristics, and EM frequency range, EMFs can produce no beneficial or harmful health effects within the human body.

A new anti-amyloid strategy by repeated REMFS not only stops cognitive impairment but also reverses it [19, 20]. In AD mice, REMFS at 915–2,000 MHz and power deposition with a specific absorption rate (SAR) of 0.25 to 5 W/kg [21, 22] stopped AD progression [23] by lowering Aβ levels [24] in numerous AD rodent studies [19–21, 25–35] without causing brain edema or hemorrhages [2, 3]. These exposures did not cause any cancer after two years of treatment [20], the main side effect was a body temperature rise (TR) of 1.3°C in the AD mice [20]. Since radiofrequency heat can cause tissue injury, it must be maintained at a safe level of less than 0.5°C, according to regulatory agencies [36]. These circumstances raised a primary question: Can a combination of variables (frequency, power, and antenna type) generate the required penetration depth with a safe and effective SAR to lower Aβ levels with a TR < 0.5°C in the human brain? Here, we will examine possible answers to develop an appropriate device for human exposure.

## Preclinical studies

### Electrical stimulation

#### Cell culture studies

Few neurostimulation studies have used cell cultures with direct current (DC) stimulation techniques like those used in clinical trials. A recent review [37] concluded that the DC stimulation effects are non-synaptic membrane polarization, are driven by glutamate, are gated by gamma-aminobutyric acid (GABA) activity reduction, require brain-derived neurotrophic factor (BDNF) expression, and require protein synthesis. In an in vitro model study, electrical stimulation did not affect the proliferation or survival of the examined cell lines but upregulated C-X-C motif chemokine 12 (CXCL12) in the astrocyte SVGA cell line and IL-1β in SH-SY5Y neuronal line [38]. Other studies found [39] that the response of the neurons to DC depended on the position and orientation of the axon and the electric parameters.

#### Animal studies

Deep brain stimulation (DBS) in animals is performed by stimulating electrodes implanted in the head, and the stimulator is connected externally, like in human DBS. Different techniques are used to apply DBS; one type is implanting the stimulators on the backs of the mice [40], and then the stimulating electrode is implanted at the target position and fixed to the skull with adhesive material. In other studies, screws are implanted in the skull to fix the electrodes [41]. In some studies, X-ray imaging or tissue staining confirms that the electrode is implanted in the target position [42]. DBS was shown to reduce Aβ plaques in the hippocampus and cortex of 6-week-old TgCRND8 mice [43] and amyloid precursor protein (APP) levels in 3×Tg mice. Also, DBS modulates glial cell activity [44] in the AD rat model.

### Magnetic stimulation

#### Cell cultures

Magnetic stimulation was applied to cell cultures derived from the frontal cortex of murine embryos; the cultures were exposed to sinus-shaped HF magnetic fields [45] to examine the effects of repetitive magnetic stimulation on gene expression. Researchers found ten significant changes in gene expression out of 171 genes using an AD-related quantitative reverse transcription-polymerase chain reaction (qRT-PCR) array. Another study applied an LF pulsed magnetic field in peripheral blood mononuclear cells obtained from AD patients. They found that magnetic field modulates the expression of proteins involved in AD, including miR-107, miR-335, miR-26b, and β-site APP-cleaving enzyme 1 (BACE1) mRNA, which would improve AD pathology. In a low-intensity static magnetic fields (SMFs) study, primary cortical and hippocampal neurons were exposed to SMF (50 G) for 7 days, and they showed a 57.1 ± 6.3% decrease in the percentage of cells experiencing etoposide-induced apoptosis, accompanied by a marked reduction in the expression of the pro-apoptotic markers [46].

#### Animal studies

Repetitive transcranial magnetic stimulation (rTMS) with a round coil at 1 Hz with 100% output reversed memory deficits in a rat model of AD. rTMS was started after 14 days post-Aβ1–42 injections in the DG area of the dorsal hippocampus bilaterally and continued for 2 weeks. rTMS reversed the decrease in BDNF and up-regulated hippocampal *N*-methyl-*D*-aspartate receptor expression, improving long-term potentiation (LTP) and spatial memory [47].

In another study, a mouse AD model was exposed to rTMS at 1 Hz or 10 Hz (30% max. output at 1.26 T) after 1 day of Aβ1–42 injections, rTMS inhibited neuronal apoptosis, activated β-catenin signaling, and increased BDNF, nerve growth factor (NGF), and doublecortin levels [48]. These positive effects were established in genetically modified rodent model studies. An APP23/PS45 mouse model of AD-like disease [49] at 1.5 months of age was exposed to LF treatment for two weeks; the treatment reversed cognitive, synaptic deficits, and LTP impairment in the hippocampal CA1 region. The underlying mechanisms likely involve reductions in BACE1 and APP processing [49]. TMS also improved spatial learning deficits and enhanced hippocampal LTP in a frequency-dependent manner in 3×Tg mice. TMS enhanced the conductance of calcium-activated potassium channels associated with cortical excitability [50].

### Repeated electromagnetic field stimulation

#### Cell culture studies

##### Animal and human immortalized cell cultures

A recent literature review [51] investigates the relationship between EMF and AD at the cellular level. Some studies show the beneficial relationship between EMF exposure and AD manifestations at the cellular level. On the contrary, some studies found no relationship with AD. For example, when IMR-32 neuroblastoma cells were exposed to 60 Hz at 50, 100, and 200 μT for four hours [52] there were no changes in the expression of APP695, an isoform of APP. In a study to assess the expression of proteins involved in the AD pathology (α3, α5, and α7 nicotinic acetylcholine receptors) in SH-SY5Y human neuroblastoma cells exposed to EMF, there was no change in expression after exposure to ELF EMF [53].

In addition, 50 Hz at 3.1 mT for 18 hours induced Aβ1–42 secretion [54], and another study showed increased production of prostaglandin E2 and decreased phagocytosis of fibrillary Aβ42 [55]. All of this demonstrates the possible harmful effects of the EMF during prolonged exposures (> 2 hours). On the other hand, short-term EMF exposures activated the Aβ clearance pathway, such as the chaperone-mediated autophagy pathway in human neuroblastoma SH-SY5Y cells, with maximum effect between 30–60 minutes of EMF exposure [56]. The ubiquitin-proteasome pathway was activated in primary hippocampal rat neurons exposed at 100 mT for 15 minutes [57]. Interestingly, when HT22 mouse hippocampal neuronal cells and SH-SY5Y human neuroblastoma cells were exposed to 1,950 MHz [58] with a high-power SAR of 6 W/kg for two hours per day for 3 days, the levels of APP, Aβ precursor protein cleaving enzyme 1, disintegrin metalloproteinase 10, and presenilin-1 were not significantly different between EMF exposed culture and controls exposures. Remarkably, this researcher previously found that a SAR of 5 W/kg was beneficial for AD pathology in rats, suggesting an upper limit for the beneficial effects on AD pathology not higher than a SAR of 5 W/kg [35].

Overall, studies investigating the effect of EMF in vitro have shown mixed results regarding the expression of genes or the level of proteins related to AD, with one study showing a decreased mRNA level of genes involved in AD, two studies showing no relationship at all, and two other studies showing an increased level of proteins involved in AD. These discrepancies in the results may be due to differences in the frequency, power deposition, or SAR, exposure period of EMF, differences in the animal model, and cell type mentioned in detail in our previous study [59]. Regulatory agencies recommend using SAR measurements for safety and radiofrequency biological effects [60].

##### Primary human cultures

In our previous study, our lab utilized primary culture because it is directly extracted from human tissue and grown in a laboratory, maintaining its natural characteristics. On the other hand, an immortalized cell line is a genetically modified cell population that can divide indefinitely in culture, often derived from a tumor and therefore different from the natural cell function [61, 62]. Previously, we found that repeated REFMS at 50 MHz, exposure times of 5, 15, 30, 60, and 120 minutes, power of 0.5 W, and a SAR of 0.6 W/kg activated the HSF [63] (master regulator of proteostasis [62] and the autophagy proteins ATG5 and ATG12 [56, 64]) in primary human fibroblasts. Given that the age-related attenuation of HSF1 [65, 66] plays a central role in the process of abnormal autophagy [67] that occurs during aging, it suggested that EMF interventions to push HSF1 toward its activated state are essential for the activation of autophagy and the clearance of abnormal proteins such as Aβ in age-related diseases [68, 69]. This prompted us to examine REMFS effects on the Aβ levels in primary human brain (PHB) cultures.

**Figure 1.**
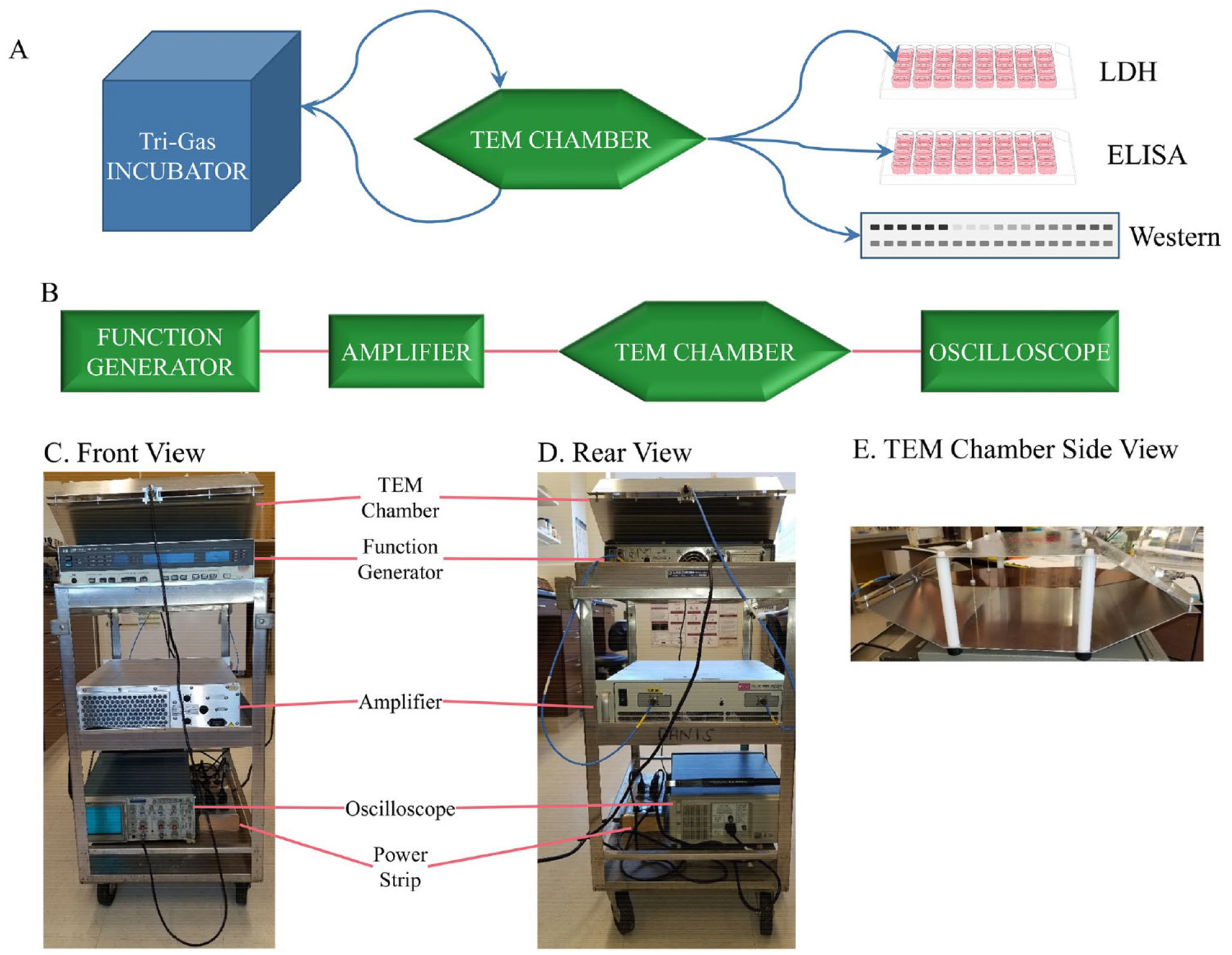
Previous REMFS experiment workflow and apparatus. (**A**) Culturing cells in a tri-gas incubator, alternating with treatments in a TEM chamber. After final treatments, cells were processed, and extracts were used for analysis. (**B**) Source of the EMF (function generator). The signal is then sent to an amplifier, then to the TEM chamber. The signal is monitored through the TEM chamber with an oscilloscope. (**C**) Front view photograph of a compact and convenient equipment system. (**D**) Rear view of the compact cart setup. (**E**) A side view of the TEM chamber shows a shelf across the middle for cell cultures. ELISA: enzyme-linked immunosorbent assay; EMF: electromagnetic field; LDH: lactate dehydrogenase; REMFS: repeated electromagnetic field stimulation; TEM: transverse electromagnetic. Reprinted from [75] (CC BY 4.0)

To find an appropriate EMF dose (dosimetry) [70], we reviewed the negative or positive actions of REMFS treatments on memory and AD pathology in multiple studies. For this purpose, we used the inverted U-shaped dose-effect curve (IUSDEC) [71]. Initially, we reviewed the literature [72–74] from cell culture [56, 63, 75–80], animal [19–21, 25–34, 81–83], and human [84–88] studies before we performed our human brain culture studies. We found that a radiofrequency power deposition that results in a SAR between 0.25–5 W/kg improves AD pathology and memory. On the contrary, when the SAR was lower than 0.25 W/kg [89–103] or higher than 5 W/kg [52, 55, 58, 104–115] it had no effects or was detrimental to AD pathology and cognition, suggesting an IUSDEC. Similarly, two human studies support an optimal SAR range. One study found impaired speed in cognitive tasks [18] at radiofrequency field radiation with a SAR of < 0.2 and > 5 W/kg, in contrast to a SAR of 1 W/kg where accuracy increased [17]. In addition, longer exposures caused demyelination in mice neurons (5 h) [116], and shorter exposures (< 30 minutes) did not have any effects [56, 59], suggesting a time and dose-dependent effect [117]. Then, following the recommendations from the International Commission on Non-Ionizing Radiation Protection (ICNIRP) [60] and IEEE [118] Standard for Safety Levels (2 W/kg local head), and considering the differences in size, geometry, tissue dielectric properties, long exposure time, thermal physiology of animals, and that neurons can be damaged even if the global SAR is within the safety limits [60], we adjusted the SAR upper limit to 0.9 W/kg and lower limit to 0.4 W/kg for our human brain cultures experiments. Therefore, we exposed PHB with different EMF frequencies, times of exposure, daily schedules, and SARs [75] to determine if REMFS was effective and safe in human brain neurons. REMFS treatment decreased Aβ40 and Aβ42 levels without evidence of toxicity. After 14 days of REMFS, we determined levels of Aβ40 peptide; treatment started on day 7 in vitro (DIV 7). Initially, we applied a frequency of 64 MHz with a SAR of 0.6 W/Kg for one hour daily for 14 days; this treatment achieved a 46% reduction in Aβ40 levels compared to the non-treated cultures [75]. Subsequently, we demonstrated that REMFS at 64 with a SAR of 0.4 W/kg for 14 days achieved a comparable reduction in Aβ40 and Aβ42 levels. Then, when we increased the exposure time from 1 to 2 hours, there was a similar reduction in the Aβ levels, so this project established the upper time limits of REMFS efficacy. We also found that a SAR of 0.4 W/kg was the minimal SAR required to lower Aβ levels, suggesting that the SAR range of 0.4–0.9 W/kg was potentially an effective and safe framework for human studies. Figure 1 demonstrates the REMFS device that was used in our previous published experiments.

#### Animal studies

Numerous studies investigated the relationship between EMF and AD in animal models; most of these are listed in recent reviews [72, 73, 119]. These take into account the various molecular biological mechanisms of AD that have been studied in various animal models, including transgenic AD mouse models and *C. elegans*, these models are explained in detail in Ribeiro et al. [120]. For example, an AD mouse model exposed to REMFS at 918 MHz with a SAR of 0.25 W/kg for two hours a day for 6 months of treatment showed an improvement in Aβ deposition and cognitive function [20]. Also, REMFS at 918 MHz in the AD mouse model produces an improvement in brain mitochondrial function and an increase in soluble Aβ1–40 following a daily two-hour exposure for a month [19]. An EMF study at 50 Hz, 10 mT for 14 days in AD rats showed improved learning and memory performance [33]. A REMFS at 1,950 MHz and SAR of 5W/kg for 2 h/day in 5×FAD mice and controls showed a significant reduction in Aβ plaques, APP, and CTFs in the hippocampus and entorhinal cortex [35]. A long-term REMFS study reduced hyperactivity and anxiety symptoms while improving memory and increased glucose metabolism [121]. Another REMFS at 918 MHz study in human and primary rat astrocytes decreased Aβ levels, reactive oxygen species (ROS), H_2_O_2_-induced phosphorylation of p38 mitogen-activated protein kinase (p38MAPK) and extracellular signal-regulated kinases 1 and 2 (ERK1/2), and mitochondrial ROS, while increasing mitochondrial membrane potential (MMP) [76]. REMFS at 1,950 MHz [22] and a SAR of 5 W/kg repressed microgliosis genes (*Csf1r*, *CD68*, and *Ccl6*), pro-inflammatory cytokine IL-1β. And microglial function genes, including *Trem2*, *Fcgr1a*, *Ctss*, and *Spi1* in 5×FAD mice, suggest that REMFS has beneficial effects in AD pathology and cognition in AD models. Several more recent REMFS studies [122–129] and systematic reviews [130–132] in rodents confirmed the beneficial effects in AD.

On the contrary, prolonged exposure to continuous EMP at 100 Hz and a very high electric field (50 kV/m), for 8 months in Sprague Dawley led to cognitive and memory impairment, increased Aβ level, increased expression of Aβ oligomer and APP, and increased expression of tau, suggesting a continuous high electric field exposure can cause harmful effects and increase AD pathology [106].

Also, another study at 100 Hz and a very high electric field (50 kV/m), for 8 months in Sprague Dawley, showed an increase in Aβ, BACE1, tau, and APP in the hippocampus, and cognitive impairment [133]. Another study at 50 Hz and low magnetic field of 100 μT in Sprague Dawley rats [134] for 12 weeks produced no effects on cognitive function and Aβ level changes, suggesting that low magnetic field does not have biological effects. Another 15-minutes REMFS exposure at 900 MHz and a higher SAR of 6 W/kg exposure showed that radiofrequency-EMF did not affect cognition in AD or control rats [135].

REMFS at 1,950 MHz and SAR 5 W/kg 2 h/day for 3 months did not improve cognition in AD mice [136], suggesting that a treatment course of 3 months does not affect AD pathology, but longer courses improve cognition and AD pathology [22, 30].

## Clinical studies

### Electrical

#### Invasive

Invasive electrical stimulation systems induce neuromodulation of dysfunctional neural circuitry [137]. These implantable neurostimulation systems target specific deep subcortical and cortical areas. These devices regulate neuron activity by using internal pulse generators to electrodes in target areas of the brain for AD, including the fornix [138], nucleus basalis of Meynert [139], and ventral capsule/ventral striatum [140].

##### Deep brain stimulation

DBS has been shown to play a role in the modulation of neural networks in AD such as the fornix that subserves memory function, specifically the Papez circuit [141].

Some alternative techniques include the ventral capsule/ventral striatum [140], which improves executive dysfunction and behavior but not memory, and the nucleus basalis of Meynert [142], which stabilized the mini-mental state exam (MMSE) and Alzheimer’s disease assessment scale-memory (ADAS-mem) but showed a decline in the ADAS-cognition (ADAS-cog). Therefore, the fornix is the most common target structure for DBS treatments, with more than one hundred patients taking this procedure [143].

In general, the results from randomized clinical trials have shown that cognitive function improved in some patients but deteriorated in others [144]. DBS takes the risk of major surgery and its complications, including bleeding, infection, pain, and hardware failure [145]. Age is an important factor in the treatment outcome. In participants below the age of 65, the ADAS-cog 13 significantly declined compared to older participants.

The proposed mechanisms of action [146] include regulation of neural networks [147], promotion of nerve oscillation [148], and reduction of Aβ [43], tau [149], and neuroinflammation [150]. It also potentially causes an increase in acetylcholine [40] and NGF in the hippocampus [151].

Although bilateral DBS appears safe for AD, evidence shows more severe complications and higher mortality events than unilateral DBS [143].

##### Vagus nerve stimulation

The surgical technique for invasive vagus nerve stimulation (iVNS) implantation is made in the cervical area 2 mm from the trachea [152]. The stimulation frequencies are between 0.5 Hz to 100 Hz and intensities from 0.6 mA to 4.5 mA. An iVNS study in AD patients showed improvement in ADAS-cog and MMSE after 24 weeks of treatment [152]. The iVNS was well tolerated, and its side effects were mild and transient.

Another iVNS study [153] found improvement in the ADAS-cog and MMSE after 6 months of treatment. Also, it showed decreased CSF-tau levels by 7.7% and cognitive improvement. In a follow-up study [154] from the previous pilot study, iVNS improved or showed no decline in the ADAS-cog in 41% of patients and the MMSE in 70% of patients after one year of treatment. Although iVNS is well tolerated, it has side effects in 10–30% of patients [155], including hematoma, superficial or deep infection, and vocal cord palsy. Post-implantation side effects are hoarseness, paresthesias, headache, and shortness of breath. In addition, technical complications [156] include lead fracture, disconnection, spontaneous turn-off, stimulator malfunction, battery or electrode failure, and lead breakage. All these complications have impeded the use of iVNS in frail patients.

#### Non-invasive

##### Transcranial direct current stimulation

Transcranial DC stimulation (tDCS) modulates the excitability thresholds of neuronal membrane potentials [157] and showed improved memory function in AD patients. A recent systematic review [158] of tDCS therapy provided a numerical evaluation of its effectiveness in improving cognitive function in AD patients. They evaluate various cognitive functions, such as memory, attention, and global cognitive function. The meta-analysis showed no effects on attention but beneficial effects on global cognitive measures and memory impairment in AD patients. They pointed out that the scarcity of trials and more high-quality studies with optimal parameters should be considered before we consider tDCS for AD treatment.

##### Transcranial alternating current stimulation

Transcranial Alternating Current Stimulation (tACS) on the other hand, provides electric current that oscillates between positive and negative values or peak-to-peak amplitude at a particular frequency [159], synchronizing the oscillations of the brain networks [160]. For instance, tACS treatments [161] on the temporal lobe for 30 days of 20 minutes per day for 6 weeks improved MMSE and ADAS-cog scores in mild to moderate AD patients.

In another study, gamma band tACS improved cognitive function MCI, but not in AD patients [162]. Follow-up after 2 years showed that non-responder MCI developed clinical disease [162]. In a recent study of combined Gamma-tACS with sound stimulation, a single patient [163] was exposed to two electrodes placed in the dorsolateral prefrontal cortex (DLPFC) and the contralateral supraorbital area, and along with sound stimulation 5 times a week for 3 weeks, there was improved global cognition.

##### Electroconvulsive treatment

Electroconvulsive Treatment (ECT) administers an electric current by a pair of electrodes, which induces a controlled seizure. A possible mechanism of action is inducing proliferative changes in the brain [164]. An ECT study showed improvement in the hippocampus after treatments [165]. Also, many studies showed improvement in depression in AD patients [166]. Another ECT study on depressed dementia, MCI group, and no cognitive impairment groups showed that MMSE scores increased after six months of treatments [167].

A retrospective cohort study of major depression patients treated with ECT reported that MMSE scores increased significantly from baseline after 6 months of therapy [168]. Moreover, AD patients have low levels of BDNF and several ECT studies showed increased levels of BDNF in depressed patients [169].

##### Cranial electrotherapy stimulation

Cranial electrotherapy stimulation (CES) devices deliver low-intensity electrical current by electrodes attached to bilateral areas of the head to modulate central and peripheral nervous system activity [170].

A CES trial stimulation with asymmetric biphasic square impulses in bursts of trains for 30 minutes per day showed significant improvement in face recognition, picture recognition, and recognition subtest of the 8-word test after 6 weeks of treatment [171] in mild AD patients. Later, a similar study compared CES with TENS found that CES improved more in the cognitive test results than TENS in mild AD patients. Moreover, a HF (100Hz) CES study showed no improvement in cognition after 6 weeks of treatment.

##### Transcutaneous vagus nerve stimulation

Transcutaneous vagus nerve stimulation (tVNS) [152] devices neurostimulate the vagus nerve by contacting the skin of the neck or the ear. tVNS was developed to circumvent the complications of the implantation of iVNS and is currently being tested in AD clinical trials. However, the tVNS has less effect due to the lack of direct stimulation of the vagus nerve. Up to now, 7 tVNS [172] trials have been tested in mild cognitive patients and not in AD patients. They have shown increased functional connectivity between the left medial prefrontal lobe and right lingual gyrus and improvement in cognition. Furthermore, connectivity from the hippocampus to several cortical and subcortical areas also demonstrated change with tVNS compared with ear lobe stimulation [173].

#### Repetitive transcranial magnetic stimulation

rTMS sends continuous magnetic pulses with the same intensity over a specific time, including LF (≤ 1 Hz) and HF (≥ 5 Hz). The LF protocol decreases excitation of the brain cortex, and the higher-frequency pulses can increase it [174]. A study of a HF (10 Hz) rTMS applied on the right inferior frontal gyrus and vertex in mild AD patients found that this stimulation led to significant improvements in attention and psychomotor speed in these patients [175]. Another study showed improvement in the UCLA auditory verbal learning test, MMSE, and the ADAS-cog after 30 sessions of HF (20 Hz) rTMS on the posterior temporal and parietal cortex for 6 weeks [176]. However, these improvements occur only when cognitive deficits are mild. Similar findings were found [177] in a study with rTMS to the bilateral DLPFC; treatment improved accuracy on action naming in 15 AD patients. Later, in a follow-up study with 24 AD patients, where researchers divided the groups according to the level of AD severity (mild to moderate and severe AD), who received rTMS over bilateral DLPFC [178] showed that both groups improved in action naming, consistent with previous findings. However, patients with early AD did not show improvement in object naming accuracy, while those with moderate to severe AD improved significantly. Furthermore, the same researchers performed an rTMS study [179] that showed higher rates of correct auditory sentence comprehension and a long-term improvement after 8 weeks of treatment.

A combined study with HF rTMS [180] with cognitive training to the bilateral DLPFC, Broca’s area, Wernicke’s area, and bilateral somatosensory cortices in mild and moderate AD patients showed that the combined approach improved the ADAS-cog and the Clinical Global Impression of Change (CGIC) scores. Another HF rTMS in early AD patients on the left DLPFC [181] for four weeks of treatment showed improved scores on the ADAS-cog in the word recall, MMSE, and Addenbrooke’s Cognitive Examination-III (ACE-III) in the attention and visual-spatial scores and these results continued for four weeks. A recent rTMS study where patients were divided into high vs. LF [182] showed that AD patients who received HF (20 Hz) rTMS on the DLPFC demonstrated a significantly higher rate of correct responses in the MMSE, and this improvement was maintained for 3 months. A HF rTMS study [136] involving bilateral DLPFC, Broca’s area, Wernicke’s area, and bilateral somatosensory cortices in patients with mild or moderate AD treated for six weeks showed improvements in ADAS-cog, MMSE, and CGIC scores, especially in the early AD patients.

On the contrary, a LF rTMS to the DLPFC in early AD demonstrated an increase in recognition memory at the end of the two-week treatment [183], and these outcomes continued for one month. In a more recent study [184], AD patients were divided into HF and LF bilateral DLPFC rTMS for two weeks; results showed lower scores on the BEHAVE-AD and ADL scores than baseline in both groups. The MMSE of HF TMS-treated patients increased from 14.22 ± 3.55 before treatment to 17.33 ± 3.11 points at 4 weeks of treatment but did not improve in the LF rTMS group.

rTMS has a beneficial on AD pathology and symptomatology. However, the main disadvantage is that the magnetic field created by the coil is transient and decays exponentially; it has a penetration depth of 2 cm under the focal region. To improve the problem of limited stimulation depth, one approach is the use of deep H-coils that allow a penetration depth of 3 cm, which is still not enough to reach important deep memory areas of the human brain [185].

#### Optogenetics and somatosensory stimulation

Optogenetics uses proteins to produce membrane potential changes by the light effects on rhodopsins of retinal cells [186]. Opsin genetic material is delivered to brain tissues via viral vectors. Opsin proteins are activated by tissue infiltration with optical fiber or direct penetration with in-sight light sources [187]. Optogenetics can provoke gamma-band oscillations (GBOs) that are abnormal findings in AD. Studies that applied GBO in hippocampal AD mice decreased Aβ plaques and induced microglia reactivity [188]. The application challenges include a reliable gene delivery system and a light source that can reach deep brain neural networks without causing tissue damage [187]. A study examined the effects of 40 Hz sound stimulation vs. non-rhythmic visual stimulation [189] in AD patients; the study improved cognition in early AD patients, in contrast with the visual stimulation which involved nature pictures displayed on a television screen.

A study that provided constant white light and noise to the control group and synchronized audiovisual stimulation at 40 Hz to the active group [190] showed decreased loss of functional connectivity, improved memory performance, and ameliorated sleep markers in the treatment group. Also, the treatment group showed more brain volume and no decline in hippocampal volume. A more recent study applied audiovisual sensory stimulation over 4 or 8 weeks with early AD [191], the study showed improved functional connectivity, cytokine levels, and immune factors in the CSF.

#### Repeated electromagnetic field stimulation

Andel et al. [192] have investigated the relationship between repeated REMFS, specifically LF, and the risk of AD. The study showed that only high levels of EMF exposure were associated with increased dementia risk in late-onset AD. Another study [193] showed no significant association between AD and EMF. Similarly, a power-frequency electromagnetic fields study that followed 2,198 individuals [194] did not find any association between the two. Conversely, other studies [195, 196] showed that REMFS increased the risk of AD mortality and the risk of AD and dementia in men. Interestingly, a study showed that exposure to extremely low intensity improves visual memory and visuo-perceptive functions in AD patients [197].

Several studies have examined the effects of EMF exposure on AD patients. In a pilot study on AD patients with cognitive impairment [198], they applied emisymmetric bilateral stimulation (EBS) at carrier wave peaks at 10.5 GHz with powers in the range of 10–100 nW for 25 minutes (3/week for 5 weeks). The treatment improved immediate and delayed memory, executive function, and behavior.

Recently, a pilot human trial [84, 85] [transcranial electromagnetic treatment (TEMT)] at 915 MHz with a SAR of 0.25 to 1.6 W/kg in eight subjects with mild-moderate AD, of which five of the original eight AD subjects completed the 2.5 year extension protocol treatment, showed a decline in the ADAS-cog in EMF treated and not treated groups, about 6 and 10 point decline respectively. This suggested that EMF treatment did not stop AD progression in humans. The main explanation for these contradictory results from the animal studies is that the frequency of 915 MHz has a penetration depth of 3.9 cm [199], unable to reach deep human brain memory areas affected early as the hippocampus, posterior cingulate, and the locus coeruleus [200–202]. Also, considering that the Aβ pathology spreads to most brain areas [203] in later stages underlies the importance of achieving an appropriate penetration depth to reach deep memory areas in a homogeneous power and SAR to prevent untreated areas or hotspots. On the other hand, EMF exposures at 64 MHz have a penetration depth of 13.5 cm [199] with a radiofrequency power deposition suitable for a human head, able to reach all deep memory areas.

**Figure 2.**
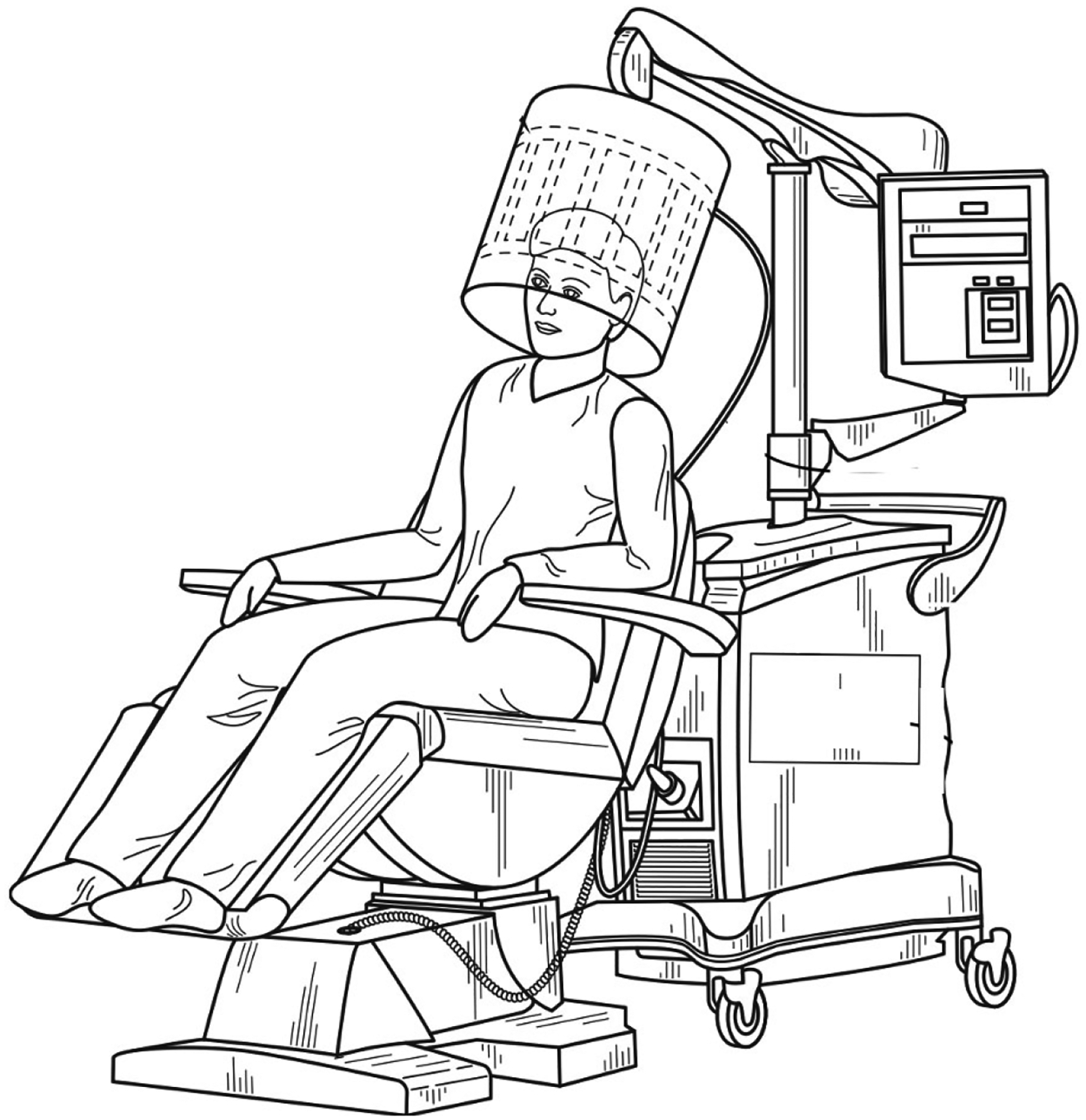
Perspective view of an REMFS with a head-mounted antenna, including control system and coupling unit. REMFS: repeated electromagnetic field stimulation. Modified with permission from “Electromagnetic Frequency Generation System and Method” by Perez, et.al. 2022. Copyright 2022 by Dr. Perez and Dr. Rizkalla (U.S. Patent Pending)

**Figure 3.**
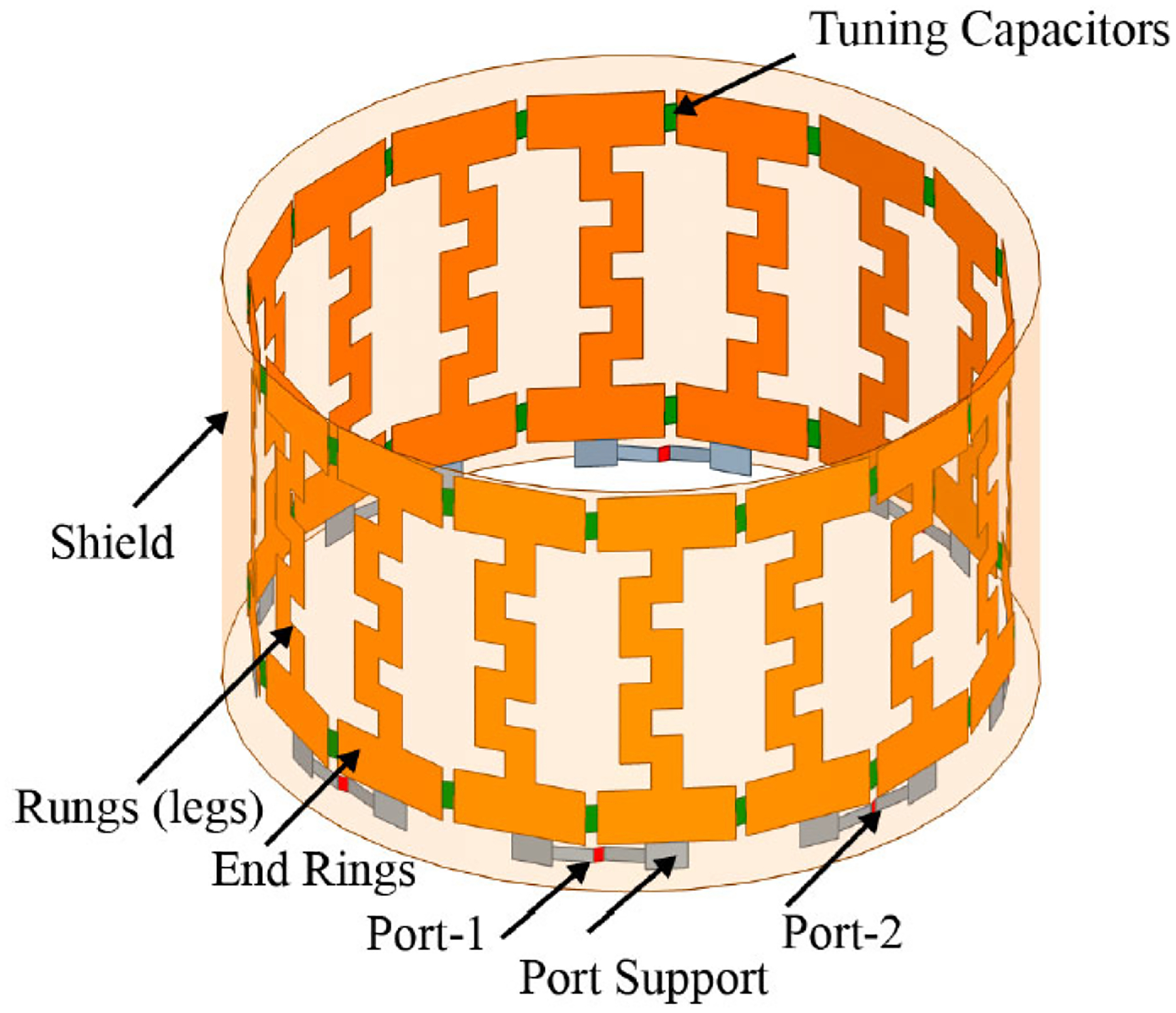
REMFS birdcage coil. The proposed REMFS birdcage coil in high-pass configuration includes 16 meander line coils and 32 tuning capacitors, with eight ports positioned at a 45° displacement from each other. REMFS: repeated electromagnetic field stimulation

Our research group has devised a patented EMF-generating system that can be used in humans. Figure 2 demonstrates a perspective view of an EMF generation system with a head-mounted antenna. It includes a control system to generate one or more time-varying electrical signals and a coupling circuit between the signal generator and head-mounted antenna to produce at least one corresponding time-varying EMF directed into the patient’s head. There is a controller to determine the power of one or more time-varying electrical signals based on premeasured physical parameters so the EMF can reach a specified depth. Figure 3 demonstrates the view of the proposed REMFS head-mounted antenna unit seen in Figure 2. It includes a Birdcage coil in high-pass configuration includes 16 meander line coils and 32 tuning capacitors, with eight ports positioned at a 45° displacement from each other. The antenna will operate at a frequency of 64 MHz, similar to routine magnetic resonance imaging (MRI).

## Limitations and future work

The future direction of this work lies in taking the success seen in numerous cellular and AD animal models and applying it to human clinical scenarios. The challenge comes in translation. The translational success of animal-to-human EMF studies can be achieved by analyzing the biological effects on animal cells or tissues caused by the power absorbed (SAR) and then utilizing computer simulations to apply the same SAR values to human tissues, considering human dielectric properties and geometry. This review has shown the varying results seen in both preclinical and clinical trials, in particular EMF therapy. Part of the discrepancy in results lies in the various parameters and instruments used by individual researchers. There is also the problem of how to account for differences in patient physical characteristics such as head size, brain structure, and tissue thickness. This is an area of active investigation, and our lab is using artificial intelligence (AI), specifically a convolutional neural network, a type of deep learning algorithm designed to analyze visual data like MRI images that will help find personalized EMF parameters. Another area of interest lies in how to determine the real-time effectiveness of REMFS for patients. New blood biomarkers such as phosphorylated tau 217 (p-tau 217) and Aβ42 and Aβ40 hold promise as potential future markers that can be monitored pre and post-EMF treatment [200]. Also, we will obtain MRI, positron emission tomography (PET) amyloid, and PET tau pre and post-treatment. Additionally, we will perform neuropsychological tests pre and post-treatment. Temperature monitoring remains an utmost safety concern as well. Since radiofrequency heat can cause tissue injury, it must be kept to a safe level of less than 0.5°C per regulatory agencies [60]. There need to be clearly defined parameters for a safe SAR level that does not exceed this threshold in all patients before investigating in human trials. AI again holds potential in this regard for calculating the temperature effects of EMF therapy while accounting for unique patient characteristics. Preclinical studies suggest that one hour of daily REMFS would be an effective and safe treatment in humans. However, clinical trials will be performed to determine the optimal and effective treatment length. Given the circadian production and clearance of Aβ [204], especially early in the disease course, this might affect the duration and timing of treatment.

## Conclusions

Here, we review all the EMF neurostimulation devices that can be used to treat AD. This review shows that the factors that prevent the development of neurostimulation as a routine include a lack of well-controlled studies, equivocal experimental results, and poor methodological standardization.

This review shows that the neurostimulation EMF devices have beneficial effects in preclinical studies. However, these treatments in humans have multiple difficulties, including differences in anatomy, geometry, tissue layers, and penetration depth. Also, some devices are invasive with the consequent risks, and others do not reach simultaneously several areas affected by AD pathology [202]. For example, rTMS is usually applied unilaterally to a localized area of the brain. When it is applied bilaterally, it causes more side effects [205], still not reaching all deep brain structures of the (2–6 cm) affected early by Aβ deposition.

In brief, the main shortcoming of the EMF experimental results regarding the biological effects of radiofrequency exposures is the consistency and inconsistencies between the results of the animal and human studies. This is explained by the difference in frequencies, penetration depth, tissue’s dielectric properties, mass density, and complex 3-D E-field distribution, all of which affect the energy absorbed or power deposition on the tissues [206, 207]. The SAR measures the energy absorbed or power deposition in the tissues relevant to specific biological effects. Therefore, it is a central consideration for EMF therapy simulations [208–211]. We cannot determine the SAR only via input power-based estimation due to its dependence on these factors. A crucial factor in the EMF and biological interaction in humans is penetration depth; the penetration depth decreases when frequency increases, which gives the skin depth within which 63% of the energy is deposited. Other important factors are the conductivity and permittivity of the tissues, as they determine how much electromagnetic radiation is absorbed by the body, with higher conductivity and permittivity leading to greater absorption; essentially, these properties dictate how readily electric fields can penetrate and interact with tissue at different frequencies, depending on the tissue type and its composition, particularly water content and ion concentration. For example, the cell phone frequency of 915 MHz has a penetration depth of 3.9 cm [199], unable to reach deep brain areas, on the other hand, the MRI frequency of 64 MHz has a penetration depth of 13.5 cm [199], able to get to important deep memory areas of the human brain. Therefore, we should consider the penetration depth of the EMF frequency before we apply it to a human brain because lower radiofrequency (10–200 MHz) have deeper penetration in a human head. Also, we should consider all the tissue layers of the human head that the EMF penetrates because most of the absorbed energy occurs in the superficial tissues. We chose 64 MHz for our studies for several reasons: A) ideal penetration depth (13.5 cm) [199] and homogeneous field distribution, B) previous studies with human cells and mouse cultures did not find toxicity [132], C) it is in the range 30–200 MHz [212] at whole human body resonance, so less power is needed to obtain SAR with a lower TR [213], and D) it has been used by MRI systems for 40 years, thus, providing an established and safe framework for human exposure.

Our review suggests that the most appropriate EMF strategy for human AD is the REMFS because it provides appropriate parameters for human treatments and addresses multiple AD issues. It can stop AD progression [4] due to anti-amyloid effects without causing bleeds or edema, and it has multitarget [5, 6] effects on beneficial molecular pathways involved in AD. Also, REMFS easily crosses the BBB [7], reaches all memory areas of the human brain, and links the aging process to AD pathology [8]. The REMFS’s initial hypothesis was that aging is the main risk factor for AD [214] and that the loss of the proteostasis [215, 216] is an early event [217] in the aging process. The loss of proteostasis is potentially the primary cause of Aβ accumulation in AD [218]. Additionally, since HSF1 has a central role in proteostasis [219], autophagy [67], Aβ clearance [220], and delaying the aging process [66, 116], this prompted us to use REMFS to activate it to lower Aβ levels. Moreover, multiple studies found that REMFS is a multitarget therapeutic strategy that activates several other beneficial AD pathways [6], including autophagy [221], the ubiquitin-proteasome system [57], oxidative stress [78], cytoprotection [79], inflammation [222], microglia activation [114], and mitochondrial and neuronal activity [19] to lower Aβ levels and potentially improve AD pathology and cognition in AD patients.

Another advantage of the REMFS technology is based on the science behind the EMF-biological systems interaction [59, 223] and the similarities with the MRI radiofrequency coil [224, 225] for its effectiveness and safety profile. The MRI coils [224, 225] apply radiofrequency pulses at similar penetration depth and SAR levels but for only a few minutes, and on the contrary, REMFS applies a longer radiofrequency continuous exposure for one hour, eventually increasing the temperature, so the device will monitor the temperature with a radiometer and an MRI-type control to adjust the power in the ports adjacent to the temperature increased. Also, the difference between MRI coils and REMFS coils is the smaller size of a portable REMFS device, which requires precise optimization. Moreover, REMFS uses 64 MHz, which provides an optimal penetration depth [226, 227] of 13.5 cm able to reach the hippocampus and other deep memory areas affected early in AD, such as the posterior cingulate and the locus coeruleus [200–202] that in later stages [228] spreads to most areas of the brain [203]. This underlies the importance of achieving an optimal penetration depth and a homogeneous power deposition with an optimal SAR to prevent untreated areas or hotspots [200, 202]. Also, the fact that REMFS uses the same frequency and power depositions with SAR values as the routine MRI that has been used for decades makes it a safe and effective strategy. Another important technical factor is that a birdcage antenna produces circular polarization necessary for biological effects [229]. Most animal studies used one antenna with polarized EMFs to activate pathways that lower Aβ and improve memory in AD mice [20] studies. EMF devices with multiple transmitters generate non-polarized EMFs with destructive or constructive interference, causing nil biological effects [229]. This polarization is an important factor in the EMF-biology interaction because radiofrequency-EMF oscillation on the H-bond causes proton tunneling [59] by increasing both vibration amplitude and the distance between the proton and acceptor at the quantum level [230], creating the protonation and conformational changes (tautomers) in RNA or other biomolecules that produce biological functions [59].

In this review, we examined the contribution of different lines of research; we used cell cultures, animal experiments, and clinical studies for the potential treatment of AD. We assessed the effectiveness and safety of REFMS and its clear implications for a broader understanding of pathophysiological mechanisms for future therapeutic interventions. Evidence demonstrated that cognitive functions affected by AD can be successfully modulated by REMFS. The lack of effective human studies makes the need for REMFS clinical trials highly significant in advancing this anti-amyloid strategy for future AD treatments.

## Abbreviations

AD: Alzheimer’s disease
ADAS: cog: Alzheimer’s disease assessment scale-cognition
AI: artificial intelligence
APP: amyloid precursor protein
Aβ: beta-amyloid
BACE1: β-site amyloid precursor protein-cleaving enzyme 1
BBB: blood-brain-barrier
BDNF: brain-derived neurotrophic factor
CGIC: Clinical Global Impression of Change
DBS: deep brain stimulation
DC: direct current
DLPFC: dorsolateral prefrontal cortex
ELF: extremely low frequency
EMF: electromagnetic field
EMPs: electromagnetic pulses
GBOs: gamma-band oscillations
HF: high frequency
IUSDEC: inverted U-shaped dose-effect curve
iVNS: invasive vagus nerve stimulation
LF: low frequency
LTP: long-term potentiation
mAbs: monoclonal antibodies
MMP: mitochondrial membrane potential
MMSE: mini-mental state exam
MRI: magnetic resonance imaging
NGF: nerve growth factor
PET: positron emission tomography
PHB: primary human brain
REMFS: repeated electromagnetic field stimulation
ROS: reactive oxygen species
rTMS: repetitive transcranial magnetic stimulation
SAR: specific absorption rate
SMFs: static magnetic fields
tDCS: transcranial direct current stimulation
TMS: transcranial magnetic stimulation
TR: temperature rise
tVNS: transcutaneous vagus nerve stimulation

## References

[1] 2022 Alzheimer’s disease facts and figures. Alzheimers Dement. 2022;18:700–89. 35289055 10.1002/alz.12638

[2] Lacorte E, Ancidoni A, Zaccaria V, Remoli G, Tariciotti L, Bellomo G, Safety and Efficacy of Monoclonal Antibodies for Alzheimer’s Disease: A Systematic Review and Meta-Analysis of Published and Unpublished Clinical Trials. J Alzheimers Dis. 2022;87:101–29. 35275549 10.3233/JAD-220046 PMC9198746

[3] Couzin-Frankel J Side effects loom over Alzheimer’s drugs. Science. 2023;381:466–7. 37535727 10.1126/science.adk0830

[4] Heidebrink JL, Paulson HL. Lessons Learned from Approval of Aducanumab for Alzheimer’s Disease. Annu Rev Med. 2024;75:99–111. 38285515 10.1146/annurev-med-051022-043645 PMC10926277

[5] Calabrò M, Rinaldi C, Santoro G, Crisafulli C. The biological pathways of Alzheimer disease: a review. AIMS Neurosci. 2020;8:86–132. 33490374 10.3934/Neuroscience.2021005 PMC7815481

[6] Iliyasu MO, Musa SA, Oladele SB, Iliya AI. Amyloid-beta aggregation implicates multiple pathways in Alzheimer’s disease: Understanding the mechanisms. Front Neurosci. 2023;17:1081938. 37113145 10.3389/fnins.2023.1081938 PMC10128090

[7] Sousa JA, Bernardes C, Bernardo-Castro S, Lino M, Albino I, Ferreira L, Reconsidering the role of blood-brain barrier in Alzheimer’s disease: From delivery to target. Front Aging Neurosci. 2023;15: 1102809. 36875694 10.3389/fnagi.2023.1102809 PMC9978015

[8] Blinkouskaya Y, Caçoilo A, Gollamudi T, Jalalian S, Weickenmeier J. Brain aging mechanisms with mechanical manifestations. Mech Ageing Dev. 2021;200:111575. 34600936 10.1016/j.mad.2021.111575 PMC8627478

[9] Hardy J, Selkoe DJ. The amyloid hypothesis of Alzheimer’s disease: progress and problems on the road to therapeutics. Science. 2002;297:353–6. 12130773 10.1126/science.1072994

[10] Canevari L, Abramov AY, Duchen MR. Toxicity of amyloid β peptide: tales of calcium, mitochondria, and oxidative stress. Neurochem Res. 2004;29:637–50. 15038611 10.1023/b:nere.0000014834.06405.af

[11] T Takada E, Okubo K, Yano Y, Iida K, Someda M, Hirasawa A, Molecular Mechanism of Apoptosis by Amyloid β-Protein Fibrils Formed on Neuronal Cells. ACS Chem Neurosci. 2020;11:796–805. 32056421 10.1021/acschemneuro.0c00011

[12] Mawuenyega KG, Sigurdson W, Ovod V, Munsell L, Kasten T, Morris JC, Decreased clearance of CNS β-amyloid in Alzheimer’s disease. Science. 2010;330:1774. 21148344 10.1126/science.1197623 PMC3073454

[13] Li Y, Rusinek H, Butler T, Glodzik L, Pirraglia E, Babich J, Decreased CSF clearance and increased brain amyloid in Alzheimer’s disease. Fluids Barriers CNS. 2022;19:21. 35287702 10.1186/s12987-022-00318-y PMC8919541

[14] Leissring MA, Farris W, Chang AY, Walsh DM, Wu X, Sun X, Enhanced proteolysis of β-amyloid in APP transgenic mice prevents plaque formation, secondary pathology, and premature death. Neuron. 2003;40:1087–93. 14687544 10.1016/s0896-6273(03)00787-6

[15] Perez FP, Bose D, Maloney B, Nho K, Shah K, Lahiri DK. Late-onset Alzheimer’s disease, heating up and foxed by several proteins: pathomolecular effects of the aging process. J Alzheimers Dis. 2014; 40:1–17. 24326519 10.3233/JAD-131544 PMC4126605

[16] Ghosn R, Yahia-Cherif L, Hugueville L, Ducorps A, Lemaréchal JD, Thuróczy G, Radiofrequency signal affects alpha band in resting electroencephalogram. J Neurophysiol. 2015;113:2753–9. 25695646 10.1152/jn.00765.2014 PMC4416621

[17] Regel SJ, Gottselig JM, Schuderer J, Tinguely G, Rétey JV, Kuster N, Pulsed radio frequency radiation affects cognitive performance and the waking electroencephalogram. Neuroreport. 2007; 18:803–7. 17471070 10.1097/WNR.0b013e3280d9435e

[18] Regel SJ, Tinguely G, Schuderer J, Adam M, Kuster N, Landolt HP, Pulsed radio-frequency electromagnetic fields: dose-dependent effects on sleep, the sleep EEG and cognitive performance. J Sleep Res. 2007;16:253–8. 17716273 10.1111/j.1365-2869.2007.00603.x

[19] Dragicevic N, Bradshaw PC, Mamcarz M, Lin X, Wang L, Cao C, Long-term electromagnetic field treatment enhances brain mitochondrial function of both Alzheimer’s transgenic mice and normal mice: a mechanism for electromagnetic field-induced cognitive benefit? Neuroscience. 2011;185: 135–49. 21514369 10.1016/j.neuroscience.2011.04.012

[20] Arendash GW, Sanchez-Ramos J, Mori T, Mamcarz M, Lin X, Runfeldt M, Electromagnetic field treatment protects against and reverses cognitive impairment in Alzheimer’s disease mice. J Alzheimers Dis. 2010;19:191–210. 20061638 10.3233/JAD-2010-1228

[21] Arendash GW, Mori T, Dorsey M, Gonzalez R, Tajiri N, Borlongan C. Electromagnetic treatment to old Alzheimer’s mice reverses β-amyloid deposition, modifies cerebral blood flow, and provides selected cognitive benefit. PLoS One. 2012;7:e35751. 22558216 10.1371/journal.pone.0035751 PMC3338462

[22] Son Y, Park HJ, Jeong YJ, Choi HD, Kim N, Lee HJ. Long-term radiofrequency electromagnetic fields exposure attenuates cognitive dysfunction in 5×FAD mice by regulating microglial function. Neural Regen Res. 2023;18:2497–503. 37282482 10.4103/1673-5374.371379 PMC10360091

[23] Bok J, Ha J, Ahn BJ, Jang Y. Disease-Modifying Effects of Non-Invasive Electroceuticals on β-Amyloid Plaques and Tau Tangles for Alzheimer’s Disease. Int J Mol Sci. 2022;24:679. 36614120 10.3390/ijms24010679 PMC9821138

[24] Cai W, Li L, Sang S, Pan X, Zhong C. Physiological Roles of β-amyloid in Regulating Synaptic Function: Implications for AD Pathophysiology. Neurosci Bull. 2023;39:1289–308. 36443453 10.1007/s12264-022-00985-9 PMC10387033

[25] Arendash GW. Transcranial electromagnetic treatment against Alzheimer’s disease: why it has the potential to trump Alzheimer’s disease drug development. J Alzheimers Dis. 2012;32:243–66. 22810103 10.3233/JAD-2012-120943

[26] Banaceur S, Banasr S, Sakly M, Abdelmelek H. Whole body exposure to 2.4 GHz WIFI signals: effects on cognitive impairment in adult triple transgenic mouse models of Alzheimer’s disease (3×Tg-AD). Behav Brain Res. 2013;240:197–201. 23195115 10.1016/j.bbr.2012.11.021

[27] Jeong YJ, Son Y, Choi HD, Kim N, Lee YS, Ko YG, Behavioral changes and gene profile alterations after chronic 1,950-MHz radiofrequency exposure: An observation in C57BL/6 mice. Brain Behav. 2020;10:e01815. 32856797 10.1002/brb3.1815 PMC7667305

[28] Kumlin T, Iivonen H, Miettinen P, Juvonen A, van Groen T, Puranen L, Mobile phone radiation and the developing brain: behavioral and morphological effects in juvenile rats. Radiat Res. 2007; 168:471–9. 17903040 10.1667/RR1002.1

[29] Wang K, Lu JM, Xing ZH, Zhao QR, Hu LQ, Xue L, Effect of 1.8 GHz radiofrequency electromagnetic radiation on novel object associative recognition memory in mice. Sci Rep. 2017;7: 44521. 28303965 10.1038/srep44521 PMC5355939

[30] Son Y, Kim JS, Jeong YJ, Jeong YK, Kwon JH, Choi HD, Long-term RF exposure on behavior and cerebral glucose metabolism in 5×FAD mice. Neurosci Lett. 2018;666:64–9. 29273398 10.1016/j.neulet.2017.12.042

[31] Hu Y, Lai J, Wan B, Liu X, Zhang Y, Zhang J, Long-term exposure to ELF-MF ameliorates cognitive deficits and attenuates tau hyperphosphorylation in 3×Tg AD mice. Neurotoxicology. 2016;53: 290–300. 26945731 10.1016/j.neuro.2016.02.012

[32] Liu X, Zuo H, Wang D, Peng R, Song T, Wang S, Improvement of spatial memory disorder and hippocampal damage by exposure to electromagnetic fields in an Alzheimer’s disease rat model. PLoS One. 2015;10:e0126963. 25978363 10.1371/journal.pone.0126963 PMC4433192

[33] Akbarnejad Z, Esmaeilpour K, Shabani M, Asadi-Shekaari M, Saeedi Goraghani M, Ahmadi-Zeidabadi M. Spatial memory recovery in Alzheimer’s rat model by electromagnetic field exposure. Int J Neurosci. 2018;128:691–6. 29185809 10.1080/00207454.2017.1411353

[34] Weisbrot D, Lin H, Ye L, Blank M, Goodman R. Effects of mobile phone radiation on reproduction and development in *Drosophila melanogaster*. J Cell Biochem. 2003;89:48–55. 12682907 10.1002/jcb.10480

[35] Jeong YJ, Kang GY, Kwon JH, Choi HD, Pack JK, Kim N, 1950 MHz Electromagnetic Fields Ameliorate Aβ Pathology in Alzheimer’s Disease Mice. Curr Alzheimer Res. 2015;12:481–92. 26017559 10.2174/156720501205150526114448 PMC5445699

[36] International Commission on Non-Ionizing Radiation Protection (ICNIRP). Guidelines for limiting exposure to time-varying electric, magnetic, and electromagnetic fields (up to 300 GHz). International Commission on Non-Ionizing Radiation Protection. Health Phys. 1998;74:494–522. 9525427

[37] Euskirchen N, Nitsche MA, van Thriel C. Direct Current Stimulation in Cell Culture Systems and Brain Slices—New Approaches for Mechanistic Evaluation of Neuronal Plasticity and Neuromodulation: State of the Art. Cells. 2021;10:3583. 34944091 10.3390/cells10123583 PMC8700319

[38] Kubelt C, Molkewehrum H, Lucius R, Synowitz M, Held-Feindt J, Helmers AK. Influence of Simulated Deep Brain Stimulation on the Expression of Inflammatory Mediators by Human Central Nervous System Cells In Vitro. Neuromolecular Med. 2022;24:169–82. 34216357 10.1007/s12017-021-08674-y PMC9117383

[39] McIntyre CC, Grill WM, Sherman DL, Thakor NV. Cellular effects of deep brain stimulation: model-based analysis of activation and inhibition. J Neurophysiol. 2004;91:1457–69. 14668299 10.1152/jn.00989.2003

[40] Huang C, Chu H, Ma Y, Zhou Z, Dai C, Huang X, The neuroprotective effect of deep brain stimulation at nucleus basalis of Meynert in transgenic mice with Alzheimer’s disease. Brain Stimul. 2019;12:161–74. 30181106 10.1016/j.brs.2018.08.015

[41] Hescham S, Lim LW, Jahanshahi A, Steinbusch HW, Prickaerts J, Blokland A, Deep brain stimulation of the forniceal area enhances memory functions in experimental dementia: the role of stimulation parameters. Brain Stimul. 2013;6:72–7. 22405739 10.1016/j.brs.2012.01.008

[42] Lee JE, Jeong DU, Lee J, Chang WS, Chang JW. The effect of nucleus basalis magnocellularis deep brain stimulation on memory function in a rat model of dementia. BMC Neurol. 2016;16:6. 26757896 10.1186/s12883-016-0529-z PMC4711102

[43] Xia F, Yiu A, Stone SSD, Oh S, Lozano AM, Josselyn SA, Entorhinal Cortical Deep Brain Stimulation Rescues Memory Deficits in Both Young and Old Mice Genetically Engineered to Model Alzheimer’s Disease. Neuropsychopharmacology. 2017;42:2493–503. 28540926 10.1038/npp.2017.100 PMC5686482

[44] Vedam-Mai V, Baradaran-Shoraka M, Reynolds BA, Okun MS. Tissue Response to Deep Brain Stimulation and Microlesion: A Comparative Study. Neuromodulation 2016;19:451–8. 27018335 10.1111/ner.12406 PMC4961567

[45] Stock M, Kirchner B, Waibler D, Cowley DE, Pfaffl MW, Kuehn R. Effect of magnetic stimulation on the gene expression profile of *in vitro* cultured neural cells. Neurosci Lett. 2012;526:122–7. 22925660 10.1016/j.neulet.2012.08.024

[46] Ben Yakir-Blumkin M, Loboda Y, Schächter L, Finberg JP. Neuroprotective effect of weak static magnetic fields in primary neuronal cultures. Neuroscience. 2014;278:313–26. 25171788 10.1016/j.neuroscience.2014.08.029

[47] Tan T, Xie J, Liu T, Chen X, Zheng X, Tong Z, Low-frequency (1Hz) repetitive transcranial magnetic stimulation (rTMS) reverses Aβ1–42-mediated memory deficits in rats. Exp Gerontol. 2013; 48:786–94. 23665072 10.1016/j.exger.2013.05.001

[48] Chen X, Chen S, Liang W, Ba F. Administration of Repetitive Transcranial Magnetic Stimulation Attenuates A*β*_1–42_-Induced Alzheimer’s Disease in Mice by Activating *β*-Catenin Signaling. Biomed Res Int. 2019;2019:1431760. 30949496 10.1155/2019/1431760 PMC6425292

[49] Huang Z, Tan T, Du Y, Chen L, Fu M, Yu Y, Low-Frequency Repetitive Transcranial Magnetic Stimulation Ameliorates Cognitive Function and Synaptic Plasticity in APP23/PS45 Mouse Model of Alzheimer’s Disease. Front Aging Neurosci. 2017;9:292. 28955219 10.3389/fnagi.2017.00292 PMC5600921

[50] Wang F, Zhang Y, Wang L, Sun P, Luo X, Ishigaki Y, Improvement of spatial learning by facilitating large-conductance calcium-activated potassium channel with transcranial magnetic stimulation in Alzheimer’s disease model mice. Neuropharmacology. 2015;97:210–9. 26051398 10.1016/j.neuropharm.2015.05.027

[51] Dasdag O, Adalier N, Dasdag S. Electromagnetic radiation and Alzheimer’s disease. Biotechnol Biotechnol Equip. 2020;34:1087–94.

[52] Rao RR, Halper J, Kisaalita WS. Effects of 60 Hz electromagnetic field exposure on APP695 transcription levels in differentiating human neuroblastoma cells. Bioelectrochemistry. 2002;57: 9–15. 12049751 10.1016/s1567-5394(02)00004-x

[53] Antonini RA, Benfante R, Gotti C, Moretti M, Kuster N, Schuderer J, Extremely low-frequency electromagnetic field (ELF-EMF) does not affect the expression of α3, α5 and α7 nicotinic receptor subunit genes in SH-SY5Y neuroblastoma cell line. Toxicol Lett. 2006;164:268–77. 16513298 10.1016/j.toxlet.2006.01.006

[54] Del Giudice E, Facchinetti F, Nofrate V, Boccaccio P, Minelli T, Dam M, Fifty Hertz electromagnetic field exposure stimulates secretion of β-amyloid peptide in cultured human neuroglioma. Neurosci Lett. 2007;418:9–12. 17382472 10.1016/j.neulet.2007.02.057

[55] He GL, Luo Z, Shen TT, Li P, Yang J, Luo X, Inhibition of STAT3- and MAPK-dependent PGE2 synthesis ameliorates phagocytosis of fibrillar β-amyloid peptide (1–42) via EP2 receptor in EMF-stimulated N9 microglial cells. J Neuroinflammation. 2016;13:296. 27871289 10.1186/s12974-016-0762-9 PMC5117690

[56] Marchesi N, Osera C, Fassina L, Amadio M, Angeletti F, Morini M, Autophagy is modulated in human neuroblastoma cells through direct exposition to low frequency electromagnetic fields. J Cell Physiol. 2014;229:1776–86. 24676932 10.1002/jcp.24631

[57] Hirai T, Taniura H, Goto Y, Ogura M, Sng JC, Yoneda Y. Stimulation of ubiquitin–proteasome pathway through the expression of amidohydrolase for N–terminal asparagine (Ntan1) in cultured rat hippocampal neurons exposed to static magnetism. J Neurochem. 2006;96:1519–30. 16539681 10.1111/j.1471-4159.2006.03655.x

[58] Park J, Kwon JH, Kim N, Song K. Effects of 1950 MHz radiofrequency electromagnetic fields on Aβ processing in human neuroblastoma and mouse hippocampal neuronal cells. J Radiat Res. 2018;59: 18–26. 29040655 10.1093/jrr/rrx045 PMC5778507

[59] Perez FP, Bandeira JP, Perez Chumbiauca CN, Lahiri DK, Morisaki J, Rizkalla M. Multidimensional insights into the repeated electromagnetic field stimulation and biosystems interaction in aging and age-related diseases. J Biomed Sci. 2022;29:39. 35698225 10.1186/s12929-022-00825-y PMC9190166

[60] International Commission on Non-Ionizing Radiation Protection (ICNIRP). Guidelines for Limiting Exposure to Electromagnetic Fields (100 kHz to 300 GHz). Health Phys. 2020;118:483–524. 32167495 10.1097/HP.0000000000001210

[61] Newton TM, Duce JA, Bayle ED. The proteostasis network provides targets for neurodegeneration. Br J Pharmacol. 2019;176:3508–14. 30820936 10.1111/bph.14643 PMC6715599

[62] Kovács D, Sigmond T, Hotzi B, Bohár B, Fazekas D, Deák V, HSF1Base: A Comprehensive Database of HSF1 (Heat Shock Factor 1) Target Genes. Int J Mol Sci. 2019;20:5815. 31752429 10.3390/ijms20225815 PMC6888953

[63] Perez FP, Zhou X, Morisaki J, Jurivich D. Electromagnetic field therapy delays cellular senescence and death by enhancement of the heat shock response. Exp Gerontol. 2008;43:307–16. 18325704 10.1016/j.exger.2008.01.004

[64] Manai F, Fassina L, Venturini L, Angeletti F, Osera C, Marchesi N, A low-frequency electromagnetic (LF-EMF) exposure scheme induces autophagy activation to counteract in vitro Abeta-amyloid neurotoxicity. Proceedings of Atti del III Convegno Nazionale Interazioni tra Campi Elettromagnetici e Biosistemi; 2014 Jul 2–4; Napoli. IRIS; 2014.

[65] Liu AY, Minetti CA, Remeta DP, Breslauer KJ, Chen KY. HSF1, Aging, and Neurodegeneration. Adv Exp Med Biol. 2023;1409:23–49. 35995906 10.1007/5584_2022_733

[66] Trivedi R, Knopf B, Rakoczy S, Manocha GD, Brown-Borg H, Jurivich DA. Disrupted HSF1 regulation in normal and exceptional brain aging. Biogerontology. 2024;25:147–60. 37707683 10.1007/s10522-023-10063-w PMC10794279

[67] Watanabe Y, Taguchi K, Tanaka M. Roles of Stress Response in Autophagy Processes and Aging-Related Diseases. Int J Mol Sci. 2023;24:13804. 37762105 10.3390/ijms241813804 PMC10531041

[68] Perez FP, Moinuddin SS, ul ain Shamim Q, Joseph DJ, Morisaki J, Zhou X. Longevity pathways: HSF1 and FoxO pathways, a new therapeutic target to prevent age-related diseases. Curr Aging Sci. 2012; 5:87–95. 21834787 10.2174/1874609811205020087

[69] Perez FP, Zhou X, Morisaki J, Ilie J, James T, Jurivich DA. Engineered repeated electromagnetic field shock therapy for cellular senescence and age-related diseases. Rejuvenation Res. 2008;11:1049–57. 19119860 10.1089/rej.2008.0793

[70] Chou CK, Bassen H, Osepchuk J, Balzano Q, Petersen R, Meltz M, Radio frequency electromagnetic exposure: tutorial review on experimental dosimetry. Bioelectromagnetics. 1996; 17:195–208. 8809359 10.1002/(SICI)1521-186X(1996)17:3<195::AID-BEM5>3.0.CO;2-Z

[71] Baldi E, Bucherelli C. The inverted “u-shaped” dose-effect relationships in learning and memory: modulation of arousal and consolidation. Nonlinearity Biol Toxicol Med. 2005;3:9–21. 19330154 10.2201/nonlin.003.01.002 PMC2657842

[72] Ahmad RHMA, Fakhoury M, Lawand N Electromagnetic Field in Alzheimer’s Disease: A Literature Review of Recent Preclinical and Clinical Studies. Curr Alzheimer Res. 2020;17:1001–12. 33256578 10.2174/1567205017666201130085853

[73] Shirbandi K, Khalafi M, J Bevelacqua J, Sadeghian N, Adiban S, Bahaeddini Zarandi F, Exposure to Low Levels of Radiofrequency Electromagnetic Fields Emitted from Cell-phones as a Promising Treatment of Alzheimer’s Disease: A Scoping Review Study. J Biomed Phys Eng. 2023;13:3–16. 36818013 10.31661/jbpe.v0i0.2109-1398 PMC9923247

[74] Biolnitiative Working Group. BioInitiative 2012: A Rationale for Biologically-based Exposure Standards for Low-Intensity Electromagnetic Radiation [Internet]. Cindy Sage, Sage Associates; c2012 [cited year month day]. Available from: https://www.centerforadvancedmed.com/wp-content/uploads/2018/10/bioInitiativeReport2012.pdf

[75] Perez FP, Maloney B, Chopra N, Morisaki JJ, Lahiri DK. Repeated electromagnetic field stimulation lowers amyloid-β peptide levels in primary human mixed brain tissue cultures. Sci Rep. 2021;11: 621. 33436686 10.1038/s41598-020-77808-2 PMC7804462

[76] Tsoy A, Saliev T, Abzhanova E, Turgambayeva A, Kaiyrlykyzy A, Akishev M, The Effects of Mobile Phone Radiofrequency Electromagnetic Fields on β-Amyloid-Induced Oxidative Stress in Human and Rat Primary Astrocytes. Neuroscience. 2019;408:46–57. 30953670 10.1016/j.neuroscience.2019.03.058

[77] Lai H, Levitt BB. Cellular and molecular effects of non-ionizing electromagnetic fields. Rev Environ Health. 2023;39:519–29. 37021652 10.1515/reveh-2023-0023

[78] Osera C, Amadio M, Falone S, Fassina L, Magenes G, Amicarelli F, Pre-exposure of neuroblastoma cell line to pulsed electromagnetic field prevents H2O2-induced ROS production by increasing MnSOD activity. Bioelectromagnetics. 2015;36:219–32. 25708841 10.1002/bem.21900

[79] Osera C, Fassina L, Amadio M, Venturini L, Buoso E, Magenes G, Cytoprotective response induced by electromagnetic stimulation on SH-SY5Y human neuroblastoma cell line. Tissue Eng Part A. 2011;17:2573–82. 21615217 10.1089/ten.TEA.2011.0071

[80] Leszczynski D, Joenväärä S, Reivinen J, Kuokka R. Non-thermal activation of the hsp27/p38MAPK stress pathway by mobile phone radiation in human endothelial cells: molecular mechanism for cancer- and blood-brain barrier-related effects. Differentiation. 2002;70:120–9. 12076339 10.1046/j.1432-0436.2002.700207.x

[81] J Jeong H, Andersson J, Hess A, Jezzard P. Effect of subject-specific head morphometry on specific absorption rate estimates in parallel-transmit MRI at 7 T. Magn Reson Med. 2023;89:2376–90. 36656151 10.1002/mrm.29589 PMC10952207

[82] Blank M, Goodman R. Electromagnetic fields stress living cells. Pathophysiology. 2009;16:71–8. 19268550 10.1016/j.pathophys.2009.01.006

[83] Shallom JM, Di Carlo AL, Ko D, Penafiel LM, Nakai A, Litovitz TA. Microwave exposure induces Hsp70 and confers protection against hypoxia in chick embryos. J Cell Biochem. 2002;86:490–6. 12210755 10.1002/jcb.10243

[84] Arendash G, Cao C, Abulaban H, Baranowski R, Wisniewski G, Becerra L, A Clinical Trial of Transcranial Electromagnetic Treatment in Alzheimer’s Disease: Cognitive Enhancement and Associated Changes in Cerebrospinal Fluid, Blood, and Brain Imaging. J Alzheimers Dis. 2019;71: 57–82. 31403948 10.3233/JAD-190367 PMC6839500

[85] Arendash G, Abulaban H, Steen S, Andel R, Wang Y, Bai Y, Transcranial Electromagnetic Treatment Stops Alzheimer’s Disease Cognitive Decline over a 2½-Year Period: A Pilot Study. Medicines (Basel). 2022;9:42. 36005647 10.3390/medicines9080042 PMC9416517

[86] Cao C, Abulaban H, Baranowski R, Wang Y, Bai Y, Lin X, Transcranial Electromagnetic Treatment “Rebalances” Blood and Brain Cytokine Levels in Alzheimer’s Patients: A New Mechanism for Reversal of Their Cognitive Impairment. Front Aging Neurosci. 2022;14:829049. 35585867 10.3389/fnagi.2022.829049 PMC9108275

[87] Söderqvist F, Hardell L, Carlberg M, Mild KH. Radiofrequency fields, transthyretin, and Alzheimer’s disease. J Alzheimers Dis. 2010;20:599–606. 20164553 10.3233/JAD-2010-1395

[88] Sandyk R Alzheimer’s disease: improvement of visual memory and visuoconstructive performance by treatment with picotesla range magnetic fields. Int J Neurosci. 1994;76:185–225. 7960477 10.3109/00207459408986003

[89] Nittby H, Widegren B, Krogh M, Grafström G, Berlin H, Rehn G, Exposure to radiation from global system for mobile communications at 1,800 MHz significantly changes gene expression in rat hippocampus and cortex. Environmentalist. 2008;28:458–65.

[90] Ntzouni MP, Stamatakis A, Stylianopoulou F, Margaritis LH. Short-term memory in mice is affected by mobile phone radiation. Pathophysiology. 2011;18:193–9. 21112192 10.1016/j.pathophys.2010.11.001

[91] Ntzouni MP, Skouroliakou A, Kostomitsopoulos N, Margaritis LH. Transient and cumulative memory impairments induced by GSM 1.8 GHz cell phone signal in a mouse model. Electromagn Biol Med. 2013;32:95–120. 23320614 10.3109/15368378.2012.709207

[92] Maaroufi K, Had-Aissouni L, Melon C, Sakly M, Abdelmelek H, Poucet B, Spatial learning, monoamines and oxidative stress in rats exposed to 900 MHz electromagnetic field in combination with iron overload. Behav Brain Res. 2014;258:80–9. 24144546 10.1016/j.bbr.2013.10.016

[93] Daniels WM, Pitout IL, Afullo TJ, Mabandla MV. The effect of electromagnetic radiation in the mobile phone range on the behaviour of the rat. Metab Brain Dis. 2009;24:629–41. 19823925 10.1007/s11011-009-9164-3

[94] Dasdag S, Akdag MZ, Kizil G, Kizil M, Cakir DU, Yokus B. Effect of 900 MHz radio frequency radiation on beta amyloid protein, protein carbonyl, and malondialdehyde in the brain. Electromagn Biol Med. 2012;31:67–74. 22268730 10.3109/15368378.2011.624654

[95] Deshmukh PS, Banerjee BD, Abegaonkar MP, Megha K, Ahmed RS, Tripathi AK, Effect of low level microwave radiation exposure on cognitive function and oxidative stress in rats. Indian J Biochem Biophys. 2013;50:114–9. 23720885

[96] Gupta SK, Mesharam MK, Krishnamurthy S. Electromagnetic radiation 2450 MHz exposure causes cognition deficit with mitochondrial dysfunction and activation of intrinsic pathway of apoptosis in rats. J Biosci. 2018;43:263–76. 29872015

[97] Megha K, Deshmukh PS, Ravi AK, Tripathi AK, Abegaonkar MP, Banerjee BD. Effect of Low-Intensity Microwave Radiation on Monoamine Neurotransmitters and Their Key Regulating Enzymes in Rat Brain. Cell Biochem Biophys. 2015;73:93–100. 25672490 10.1007/s12013-015-0576-x

[98] Megha K, Deshmukh PS, Banerjee BD, Tripathi AK, Abegaonkar MP. Microwave radiation induced oxidative stress, cognitive impairment and inflammation in brain of Fischer rats. Indian J Exp Biol. 2012;50:889–96. 23986973

[99] Tang J, Zhang Y, Yang L, Chen Q, Tan L, Zuo S, Exposure to 900 MHz electromagnetic fields activates the mkp-1/ERK pathway and causes blood-brain barrier damage and cognitive impairment in rats. Brain Res. 2015;1601:92–101. 25598203 10.1016/j.brainres.2015.01.019

[100] Kim JY, Kim HJ, Kim N, Kwon JH, Park MJ. Effects of radiofrequency field exposure on glutamate-induced oxidative stress in mouse hippocampal HT22 cells. Int J Radiat Biol. 2017;93:249–56. 27648632 10.1080/09553002.2017.1237058

[101] Jorge-Mora T, Folgueiras MA, Leiro-Vidal JM, Jorge-Barreiro F, Ares-Pena F, Lopez-Martin ME. Exposure to 2.45 GHz microwave radiation provokes cerebral changes in induction of HSP-90 α/β heat shock protein in rat. Prog Electromagn Res. 2010;100:351–79.

[102] Stefi AL, Margaritis LH, Skouroliakou AS, Vassilacopoulou D. Mobile phone electromagnetic radiation affects Amyloid Precursor Protein and α-synuclein metabolism in SH-SY5Y cells. Pathophysiology. 2019;26:203–12. 30850244 10.1016/j.pathophys.2019.02.004

[103] Aslan A, İkinci A, Baş O, Sönmez OF, Kaya H, Odacı E. Long-term exposure to a continuous 900 MHz electromagnetic field disrupts cerebellar morphology in young adult male rats. Biotech Histochem. 2017;92:324–30. 28506085 10.1080/10520295.2017.1310295

[104] Verma S, Keshri GK, Karmakar S, Mani KV, Chauhan S, Yadav A, Effects of Microwave 10 GHz Radiation Exposure in the Skin of Rats: An Insight on Molecular Responses. Radiat Res. 2021;196: 404–16. 34407201 10.1667/RADE-20-00155.1

[105] Barthélémy A, Mouchard A, Bouji M, Blazy K, Puigsegur R, Villégier AS. Glial markers and emotional memory in rats following acute cerebral radiofrequency exposures. Environ Sci Pollut Res Int. 2016; 23:25343–55. 27696165 10.1007/s11356-016-7758-y

[106] Jiang DP, Li J, Zhang J, Xu SL, Kuang F, Lang HY, Electromagnetic pulse exposure induces overexpression of beta amyloid protein in rats. Arch Med Res. 2013;44:178–84. 23523687 10.1016/j.arcmed.2013.03.005

[107] Jiang DP, Li JH, Zhang J, Xu SL, Kuang F, Lang HY, Long-term electromagnetic pulse exposure induces Abeta deposition and cognitive dysfunction through oxidative stress and overexpression of APP and BACE1. Brain Res. 2016;1642:10–9. 26972535 10.1016/j.brainres.2016.02.053

[108] Qiao S, Peng R, Yan H, Gao Y, Wang C, Wang S, Reduction of phosphorylated synapsin I (ser-553) leads to spatial memory impairment by attenuating GABA release after microwave exposure in Wistar rats. PLoS One. 2014;9:e95503. 24743689 10.1371/journal.pone.0095503 PMC3990695

[109] Prochnow N, Gebing T, Ladage K, Krause-Finkeldey D, El Ouardi A, Bitz A, Electromagnetic field effect or simply stress? Effects of UMTS exposure on hippocampal longterm plasticity in the context of procedure related hormone release. PLoS One. 2011;6:e19437. 21573218 10.1371/journal.pone.0019437 PMC3088670

[110] Wang H, Peng R, Zhou H, Wang S, Gao Y, Wang L, Impairment of long-term potentiation induction is essential for the disruption of spatial memory after microwave exposure. Int J Radiat Biol. 2013;89:1100–7. 23786183 10.3109/09553002.2013.817701

[111] Wang H, Peng R, Zhao L, Wang S, Gao Y, Wang L, The relationship between NMDA receptors and microwave-induced learning and memory impairment: a long-term observation on Wistar rats. Int J Radiat Biol. 2015;91:262–9. 25426698 10.3109/09553002.2014.988893

[112] Wang H, Tan S, Xu X, Zhao L, Zhang J, Yao B, Long term impairment of cognitive functions and alterations of NMDAR subunits after continuous microwave exposure. Physiol Behav. 2017;181:1–9. 28866028 10.1016/j.physbeh.2017.08.022

[113] Foroozandeh E, Ahadi H, Askari P. Effects of 90min Exposure to 8mT Electromagnetic Fields on Memory in Mice. J Am Sci. 2011;7:58–61.

[114] Yang X, He G, Hao Y, Chen C, Li M, Wang Y, The role of the JAK2-STAT3 pathway in pro-inflammatory responses of EMF-stimulated N9 microglial cells. J Neuroinflammation. 2010;7:54. 20828402 10.1186/1742-2094-7-54 PMC2945324

[115] Cleary SF, Cao G, Liu LM, Egle PM, Shelton KR. Stress proteins are not induced in mammalian cells exposed to radiofrequency or microwave radiation. Bioelectromagnetics. 1997;18:499–505. 9338631 10.1002/(sici)1521-186x(1997)18:7<499::aid-bem5>3.0.co;2-y

[116] Kim JH, Yu DH, Huh YH, Lee EH, Kim HG, Kim HR. Long-term exposure to 835 MHz RF-EMF induces hyperactivity, autophagy and demyelination in the cortical neurons of mice. Sci Rep. 2017;7:41129. 28106136 10.1038/srep41129 PMC5247706

[117] Maalouf J, Pelletier A, Corona A, Gay-Quéheillard J, Bach V, de Seze R, Dose- and Time-Dependent Effects of Radiofrequency Electromagnetic Field on Adipose Tissue: Implications of Thermoregulation and Mitochondrial Signaling. Int J Mol Sci. 2023;24:10628. 37445806 10.3390/ijms241310628 PMC10342026

[118] Bailey WH, Bodemann R, Bushberg J, Chou CK, Cleveland R, Faraone A. Synopsis of IEEE Std C95.1^™^-2019 “IEEE Standard for Safety Levels With Respect to Human Exposure to Electric, Magnetic, and Electromagnetic Fields, 0 Hz to 300 GHz. IEEE Access. 2019;7:171346–56.

[119] Lanni I, Chiacchierini G, Papagno C, Santangelo V, Campolongo P. Treating Alzheimer’s disease with brain stimulation: From preclinical models to non-invasive stimulation in humans. Neurosci Biobehav Rev. 2024;165:105831. 39074672 10.1016/j.neubiorev.2024.105831

[120] Ribeiro FM, Camargos ER, de Souza LC, Teixeira AL. Animal models of neurodegenerative diseases. Braz J Psychiatry. 2013;35:S82–91. 24271230 10.1590/1516-4446-2013-1157

[121] Guerriero F, Botarelli E, Mele G, Polo L, Zoncu D, Renati P, An innovative intervention for the treatment of cognitive impairment-Emisymmetric bilateral stimulation improves cognitive functions in Alzheimer’s disease and mild cognitive impairment: an open-label study. Neuropsychiatr Dis Treat. 2015;11:2391–404. 26425094 10.2147/NDT.S90966 PMC4581783

[122] Zhi W, Zou Y, Ma L, He S, Guo Z, Zhao X, 900 MHZ electromagnetic field exposure relieved AD-like symptoms on APP/PS1 mice: A potential non-invasive strategy for AD treatment. Biochem Biophys Res Commun. 2023;658:97–106. 37030070 10.1016/j.bbrc.2023.03.083

[123] Komaki A, Salehi I, Keymoradzadeh A, Taheri Azandaryani M, Golipoor Z. Effect of Long-term Exposure to Extremely Low-frequency Electromagnetic Fields on β-amyloid Deposition and Microglia Cells in an Alzheimer Model in Rats. JGUMS. 2021;30:218–29.

[124] Zhang S, Wu XN, Li Z, Mu YZ, Shu X, Zhou Q, Effects of 2.4 GHz radiofrequency electromagnetic field exposure on hippocampal proteins in APP/PS1 mice. Res Square. 2024.

[125] Teranishi M, Ito M, Huang Z, Nishiyama Y, Masuda A, Mino H, Extremely Low-Frequency Electromagnetic Field (ELF-EMF) Increases Mitochondrial Electron Transport Chain Activities and Ameliorates Depressive Behaviors in Mice. Int J Mol Sci. 2024;25:11315. 39457098 10.3390/ijms252011315 PMC11508854

[126] Kim JH, Yu DH, Kim HJ, Huh YH, Cho SW, Lee JK, Exposure to 835 MHz radiofrequency electromagnetic field induces autophagy in hippocampus but not in brain stem of mice. Toxicol Ind Health. 2018;34:23–35. 29166827 10.1177/0748233717740066

[127] Guo RW, Xie WJ, Yu B, Song C, Ji XM, Wang XY, Rotating magnetic field inhibits Aβ protein aggregation and alleviates cognitive impairment in Alzheimer’s disease mice. Zool Res. 2024;45: 924–36. 39021081 10.24272/j.issn.2095-8137.2024.034 PMC11298676

[128] Zhang J, Chen Y, Zhao Y, Wang P, Ding H, Liu C, Terahertz Irradiation Improves Cognitive Impairments and Attenuates Alzheimer’s Neuropathology in the APPSWE/PS1DE9 Mouse: A Novel Therapeutic Intervention for Alzheimer’s Disease. Neurosci Bull. 2024;40:857–71. 37971654 10.1007/s12264-023-01145-3 PMC11250709

[129] Moya-Gómez A, Font LP, Burlacu A, Alpizar YA, Cardonne MM, Brône B, Extremely Low-Frequency Electromagnetic Stimulation (ELF-EMS) Improves Neurological Outcome and Reduces Microglial Reactivity in a Rodent Model of Global Transient Stroke. Int J Mol Sci. 2023;24:11117. 37446295 10.3390/ijms241311117 PMC10342400

[130] Abkhezr H, Mohaddes G, Nikniaz Z, Farhangi MA, Heydari H, Nikniaz L. The effect of extremely low frequency electromagnetic field on spatial memory of mice and rats: a systematic review. Learn Motiv. 2023;81:101873.

[131] Eskandani R, Zibaii MI. Unveiling the biological effects of radio-frequency and extremely-low frequency electromagnetic fields on the central nervous system performance. Bioimpacts. 2024;14: 30064. 39104617 10.34172/bi.2023.30064 PMC11298025

[132] Perez FP, Morisaki J, Kanakri H, Rizkalla M. Electromagnetic Field Stimulation Therapy for Alzheimer’s Disease. Neurology (Chic). 2024;3:1020. 38699565 PMC11064876

[133] Kim JH, Lee JK, Kim HG, Kim KB, Kim HR. Possible Effects of Radiofrequency Electromagnetic Field Exposure on Central Nerve System. Biomol Ther (Seoul). 2019;27:265–75. 30481957 10.4062/biomolther.2018.152 PMC6513191

[134] Zhang Y, Liu X, Zhang J, Li N. Short-term effects of extremely low frequency electromagnetic fields exposure on Alzheimer’s disease in rats. Int J Radiat Biol. 2015;91:28–34. 25118893 10.3109/09553002.2014.954058

[135] Bouji M, Lecomte A, Gamez C, Blazy K, Villégier AS. Impact of Cerebral Radiofrequency Exposures on Oxidative Stress and Corticosterone in a Rat Model of Alzheimer’s Disease. J Alzheimers Dis. 2020; 73:467–76. 31796670 10.3233/JAD-190593

[136] Son Y, Jeong YJ, Kwon JH, Choi HD, Pack JK, Kim N, 1950 MHz radiofrequency electromagnetic fields do not aggravate memory deficits in 5×FAD mice. Bioelectromagnetics. 2016;37:391–9. 27434853 10.1002/bem.21992 PMC5108492

[137] Edwards CA, Kouzani A, Lee KH, Ross EK. Neurostimulation Devices for the Treatment of Neurologic Disorders. Mayo Clin Proc. 2017;92:1427–44. 28870357 10.1016/j.mayocp.2017.05.005

[138] Li R, Zhang C, Rao Y, Yuan TF. Deep brain stimulation of fornix for memory improvement in Alzheimer’s disease: A critical review. Ageing Res Rev. 2022;79:101668. 35705176 10.1016/j.arr.2022.101668

[139] Chen YS, Shu K, Kang HC. Deep Brain Stimulation in Alzheimer’s Disease: Targeting the Nucleus Basalis of Meynert. J Alzheimers Dis. 2021;80:53–70. 33492288 10.3233/JAD-201141

[140] Scharre DW, Weichart E, Nielson D, Zhang J, Agrawal P, Sederberg PB, Deep Brain Stimulation of Frontal Lobe Networks to Treat Alzheimer’s Disease. J Alzheimers Dis. 2018;62:621–33. 29400666 10.3233/JAD-170082

[141] Miller JP, Sweet JA, Bailey CM, Munyon CN, Luders HO, Fastenau PS. Visual-spatial memory may be enhanced with theta burst deep brain stimulation of the fornix: a preliminary investigation with four cases. Brain. 2015;138:1833–42. 26106097 10.1093/brain/awv095

[142] Baldermann JC, Hardenacke K, Hu X, Köster P, Horn A, Freund HJ, Neuroanatomical Characteristics Associated With Response to Deep Brain Stimulation of the Nucleus Basalis of Meynert for Alzheimer’s Disease. Neuromodulation. 2018;21:184–90. 28653404 10.1111/ner.12626

[143] Bittlinger M, Müller S. Opening the debate on deep brain stimulation for Alzheimer disease - a critical evaluation of rationale, shortcomings, and ethical justification. BMC Med Ethics. 2018;19:41. 29886845 10.1186/s12910-018-0275-4 PMC5994654

[144] Ríos AS, Oxenford S, Neudorfer C, Butenko K, Li N, Rajamani N, Optimal deep brain stimulation sites and networks for stimulation of the fornix in Alzheimer’s disease. Nat Commun. 2022;13:7707. 36517479 10.1038/s41467-022-34510-3 PMC9751139

[145] Barrett K Psychiatric neurosurgery in the 21st century: overview and the growth of deep brain stimulation. BJPsych Bull. 2017;41:281–6. 29018554 10.1192/pb.bp.116.055772 PMC5623888

[146] Luo Y, Sun Y, Tian X, Zheng X, Wang X, Li W, Deep Brain Stimulation for Alzheimer’s Disease: Stimulation Parameters and Potential Mechanisms of Action. Front Aging Neurosci. 2021;13:619543. 33776742 10.3389/fnagi.2021.619543 PMC7990787

[147] Hamani C, McAndrews MP, Cohn M, Oh M, Zumsteg D, Shapiro CM, Memory enhancement induced by hypothalamic/fornix deep brain stimulation. Ann Neurol. 2008;63:119–23. 18232017 10.1002/ana.21295

[148] Senova S, Chaillet A, Lozano AM. Fornical Closed-Loop Stimulation for Alzheimer’s Disease. Trends Neurosci. 2018;41:418–28. 29735372 10.1016/j.tins.2018.03.015

[149] Mann A, Gondard E, Tampellini D, Milsted JAT, Marillac D, Hamani C, Chronic deep brain stimulation in an Alzheimer’s disease mouse model enhances memory and reduces pathological hallmarks. Brain Stimul. 2018;11:435–44. 29246746 10.1016/j.brs.2017.11.012

[150] Leplus A, Lauritzen I, Melon C, Kerkerian-Le Goff L, Fontaine D, Checler F. Chronic fornix deep brain stimulation in a transgenic Alzheimer’s rat model reduces amyloid burden, inflammation, and neuronal loss. Brain Struct Funct. 2019;224:363–72. 30341742 10.1007/s00429-018-1779-x

[151] Hardenacke K, Kuhn J, Lenartz D, Maarouf M, Mai JK, Bartsch C, Stimulate or degenerate: deep brain stimulation of the nucleus basalis Meynert in Alzheimer dementia. World Neurosurg. 2013;80: S27.e35–43. 10.1016/j.wneu.2012.12.005 23246738

[152] Vargas-Caballero M, Warming H, Walker R, Holmes C, Cruickshank G, Patel B. Vagus Nerve Stimulation as a Potential Therapy in Early Alzheimer’s Disease: A Review. Front Hum Neurosci. 2022;16:866434. 35572001 10.3389/fnhum.2022.866434 PMC9098960

[153] Merrill CA, Bunker M. P3–032 Effects of vagus nerve stimulation on cognition, CSF-Tau and cerebral blood flow in patients with Alzheimer’s disease: results of a 1 year pilot study. Neurobiol Aging. 2004;25:S360.

[154] Merrill CA, Jonsson MA, Minthon L, Ejnell H, C-son Silander H, Blennow K, Vagus nerve stimulation in patients with Alzheimer’s disease: Additional follow-up results of a pilot study through 1 year. J Clin Psychiatry. 2006;67:1171–8. 16965193 10.4088/jcp.v67n0801

[155] Morris GL 3rd, Mueller WM. Long-term treatment with vagus nerve stimulation in patients with refractory epilepsy. Neurology. 1999;53:1731–5. 10563620 10.1212/wnl.53.8.1731

[156] Kahlow H, Olivecrona M. Complications of vagal nerve stimulation for drug-resistant epilepsy: a single center longitudinal study of 143 patients. Seizure. 2013;22:827–33. 23867218 10.1016/j.seizure.2013.06.011

[157] Kuo MF, Nitsche MA. Effects of transcranial electrical stimulation on cognition. Clin EEG Neurosci. 2012;43:192–9. 22956647 10.1177/1550059412444975

[158] Majdi A, van Boekholdt L, Sadigh-Eteghad S, Mc Laughlin M. A systematic review and meta-analysis of transcranial direct-current stimulation effects on cognitive function in patients with Alzheimer’s disease. Mol Psychiatry. 2022;27:2000–9. 35115703 10.1038/s41380-022-01444-7

[159] Chaieb L, Antal A, Paulus W. Transcranial alternating current stimulation in the low kHz range increases motor cortex excitability. Restor Neurol Neurosci. 2011;29:167–75. 21586823 10.3233/RNN-2011-0589

[160] Reato D, Rahman A, Bikson M, Parra LC. Effects of weak transcranial alternating current stimulation on brain activity—a review of known mechanisms from animal studies. Front Hum Neurosci. 2013; 7:687. 24167483 10.3389/fnhum.2013.00687 PMC3805939

[161] Zhou D, Li A, Li X, Zhuang W, Liang Y, Zheng CY, Effects of 40 Hz transcranial alternating current stimulation (tACS) on cognitive functions of patients with Alzheimer’s disease: a randomised, double-blind, sham-controlled clinical trial. J Neurol Neurosurg Psychiatry. 2022;93:568–70. 34764150 10.1136/jnnp-2021-326885

[162] Naro A, Corallo F, De Salvo S, Marra A, Di Lorenzo G, Muscarà N, Promising Role of Neuromodulation in Predicting the Progression of Mild Cognitive Impairment to Dementia. J Alzheimers Dis. 2016;53:1375–88. 27392866 10.3233/JAD-160305

[163] Yang QX, Wang J, Zhang X, Collins CM, Smith MB, Liu H, Analysis of wave behavior in lossy dielectric samples at high field. Magn Reson Med. 2002;47:982–9. 11979578 10.1002/mrm.10137

[164] Madsen TM, Treschow A, Bengzon J, Bolwig TG, Lindvall O, Tingström A. Increased neurogenesis in a model of electroconvulsive therapy. Biol Psychiatry. 2000;47:1043–9. 10862803 10.1016/s0006-3223(00)00228-6

[165] Bouckaert F, De Winter FL, Emsell L, Dols A, Rhebergen D, Wampers M, Grey matter volume increase following electroconvulsive therapy in patients with late life depression: a longitudinal MRI study. J Psychiatry Neurosci. 2016;41:105–14. 26395813 10.1503/jpn.140322 PMC4764479

[166] Burke AD, Goldfarb D, Bollam P, Khokher S. Diagnosing and Treating Depression in Patients with Alzheimer’s Disease. Neurol Ther. 2019;8:325–50. 31435870 10.1007/s40120-019-00148-5 PMC6858899

[167] Hausner L, Damian M, Sartorius A, Frölich L. Efficacy and cognitive side effects of electroconvulsive therapy (ECT) in depressed elderly inpatients with coexisting mild cognitive impairment or dementia. J Clin Psychiatry. 2011;72:91–7. 21208587 10.4088/JCP.10m05973gry

[168] Fernie G, Bennett DM, Currie J, Perrin JS, Reid IC. Detecting objective and subjective cognitive effects of electroconvulsive therapy: intensity, duration and test utility in a large clinical sample. Psychol Med. 2014;44:2985–94. 25065412 10.1017/S0033291714000658

[169] Rocha RB, Dondossola ER, Grande AJ, Colonetti T, Ceretta LB, Passos IC, Increased BDNF levels after electroconvulsive therapy in patients with major depressive disorder: A meta-analysis study. J Psychiatr Res. 2016;83:47–53. 27552533 10.1016/j.jpsychires.2016.08.004

[170] Chang CH, Lane HY, Lin CH. Brain Stimulation in Alzheimer’s Disease. Front Psychiatry. 2018;9:201. 29910746 10.3389/fpsyt.2018.00201 PMC5992378

[171] Scherder EJ, Bouma A, Steen AM. Effects of short-term transcutaneous electrical nerve stimulation on memory and affective behaviour in patients with probable Alzheimer’s disease. Behav Brain Res. 1995;67:211–9. 7779292 10.1016/0166-4328(94)00115-v

[172] Kamoga R, Rukundo GZ, Kalungi S, Adriko W, Nakidde G, Obua C, Vagus nerve stimulation in dementia: A scoping review of clinical and pre-clinical studies. AIMS Neurosci. 2024;11:398–420. 39431268 10.3934/Neuroscience.2024024 PMC11486617

[173] Murphy AJ, O’Neal AG, Cohen RA, Lamb DG, Porges EC, Bottari SA, The Effects of Transcutaneous Vagus Nerve Stimulation on Functional Connectivity Within Semantic and Hippocampal Networks in Mild Cognitive Impairment. Neurotherapeutics. 2023;20:419–30. 36477709 10.1007/s13311-022-01318-4 PMC10121945

[174] Sandrini M, Umiltà C, Rusconi E. The use of transcranial magnetic stimulation in cognitive neuroscience: a new synthesis of methodological issues. Neurosci Biobehav Rev. 2011;35:516–36. 20599555 10.1016/j.neubiorev.2010.06.005

[175] Eliasova I, Anderkova L, Marecek R, Rektorova I. Non-invasive brain stimulation of the right inferior frontal gyrus may improve attention in early Alzheimer’s disease: a pilot study. J Neurol Sci. 2014; 346:318–22. 25216556 10.1016/j.jns.2014.08.036

[176] Zhao J, Li Z, Cong Y, Zhang J, Tan M, Zhang H, Repetitive transcranial magnetic stimulation improves cognitive function of Alzheimer’s disease patients. Oncotarget. 2017;8:33864–71. 27823981 10.18632/oncotarget.13060 PMC5464918

[177] Cotelli M, Manenti R, Cappa SF, Geroldi C, Zanetti O, Rossini PM, Effect of transcranial magnetic stimulation on action naming in patients with Alzheimer disease. Arch Neurol. 2006;63:1602–4. 17101829 10.1001/archneur.63.11.1602

[178] Cotelli M, Manenti R, Cappa SF, Zanetti O, Miniussi C. Transcranial magnetic stimulation improves naming in Alzheimer disease patients at different stages of cognitive decline. Eur J Neurol. 2008;15: 1286–92. 19049544 10.1111/j.1468-1331.2008.02202.x

[179] Cotelli M, Manenti R, Rosini S, Calabria M, Brambilla M, Bisiacchi PS, Action and Object Naming in Physiological Aging: An rTMS Study. Front Aging Neurosci. 2010;2:151. 21151376 10.3389/fnagi.2010.00151 PMC2996246

[180] Bentwich J, Dobronevsky E, Aichenbaum S, Shorer R, Peretz R, Khaigrekht M, Beneficial effect of repetitive transcranial magnetic stimulation combined with cognitive training for the treatment of Alzheimer’s disease: a proof of concept study. J Neural Transm (Vienna). 2011;118:463–71. 21246222 10.1007/s00702-010-0578-1

[181] Zhang JJQ, Fong KNK, Ouyang RG, Siu AMH, Kranz GS. Effects of repetitive transcranial magnetic stimulation (rTMS) on craving and substance consumption in patients with substance dependence: a systematic review and meta-analysis. Addiction. 2019;114:2137–49. 31328353 10.1111/add.14753

[182] Ahmed MA, Darwish ES, Khedr EM, El Serogy YM, Ali AM. Effects of low versus high frequencies of repetitive transcranial magnetic stimulation on cognitive function and cortical excitability in Alzheimer’s dementia. J Neurol. 2012;259:83–92. 21671144 10.1007/s00415-011-6128-4

[183] Turriziani P, Smirni D, Mangano GR, Zappalà G, Giustiniani A, Cipolotti L, Low-Frequency Repetitive Transcranial Magnetic Stimulation of the Right Dorsolateral Prefrontal Cortex Enhances Recognition Memory in Alzheimer’s Disease. J Alzheimers Dis. 2019;72:613–22. 31609693 10.3233/JAD-190888

[184] Jiang W, Wu Z, Wen L, Sun L, Zhou M, Jiang X, The Efficacy of High- or Low-Frequency Transcranial Magnetic Stimulation in Alzheimer’s Disease Patients with Behavioral and Psychological Symptoms of Dementia. Adv Ther. 2022;39:286–95. 34716559 10.1007/s12325-021-01964-8

[185] Deng ZD, Lisanby SH, Peterchev AV. Electric field depth-focality tradeoff in transcranial magnetic stimulation: simulation comparison of 50 coil designs. Brain Stimul. 2013;6:1–13. 22483681 10.1016/j.brs.2012.02.005 PMC3568257

[186] Cho WH, Barcelon E, Lee SJ. Optogenetic Glia Manipulation: Possibilities and Future Prospects. Exp Neurobiol. 2016;25:197–204. 27790054 10.5607/en.2016.25.5.197 PMC5081466

[187] Mirzayi P, Shobeiri P, Kalantari A, Perry G, Rezaei N. Optogenetics: implications for Alzheimer’s disease research and therapy. Mol Brain. 2022;15:20. 35197102 10.1186/s13041-022-00905-y PMC8867657

[188] Iaccarino HF, Singer AC, Martorell AJ, Rudenko A, Gao F, Gillingham TZ, Gamma frequency entrainment attenuates amyloid load and modifies microglia. Nature. 2016;540:230–5. 27929004 10.1038/nature20587 PMC5656389

[189] Clements-Cortes A, Ahonen H, Evans M, Freedman M, Bartel L. Short-Term Effects of Rhythmic Sensory Stimulation in Alzheimer’s Disease: An Exploratory Pilot Study. J Alzheimers Dis. 2016;52: 651–60. 27031491 10.3233/JAD-160081

[190] Chan D, Suk HJ, Jackson BL, Milman NP, Stark D, Klerman EB, Gamma frequency sensory stimulation in probable mild Alzheimer’s dementia patients: results of a preliminary clinical trial. Cell Press J Sneak Peek. 2021.

[191] He Q, Colon-Motas KM, Pybus AF, Piendel L, Seppa JK, Walker ML, A feasibility trial of gamma sensory flicker for patients with prodromal Alzheimer’s disease. Alzheimers Dement (N Y). 2021;7: e12178. 34027028 10.1002/trc2.12178 PMC8118113

[192] Andel R, Crowe M, Feychting M, Pedersen NL, Fratiglioni L, Johansson B, Work-related exposure to extremely low-frequency magnetic fields and dementia: results from the population-based study of dementia in Swedish twins. J Gerontol A Biol Sci Med Sci. 2010;65:1220–7. 20622138 10.1093/gerona/glq112 PMC2954236

[193] Seidler A, Geller P, Nienhaus A, Bernhardt T, Ruppe I, Eggert S, Occupational exposure to low frequency magnetic fields and dementia: a case-control study. Occup Environ Med. 2007;64:108–14. 17043077 10.1136/oem.2005.024190 PMC2078432

[194] Li CY, Sung FC, Wu SC. Risk of cognitive impairment in relation to elevated exposure to electromagnetic fields. J Occup Environ Med. 2002;44:66–72. 11802468 10.1097/00043764-200201000-00011

[195] Gunnarsson LG, Bodin L. Occupational Exposures and Neurodegenerative Diseases—A Systematic Literature Review and Meta-Analyses. Int J Environ Res Public Health. 2019;16:337. 30691095 10.3390/ijerph16030337 PMC6388365

[196] Sobel E, Davanipour Z. Electromagnetic field exposure may cause increased production of amyloid beta and eventually lead to Alzheimer’s disease. Neurology. 1996;47:1594–600. 8960756 10.1212/wnl.47.6.1594

[197] Nystuen KL, McNamee SM, Akula M, Holton KM, DeAngelis MM, Haider NB. Alzheimer’s Disease: Models and Molecular Mechanisms Informing Disease and Treatments. Bioengineering (Basel). 2024;11:45. 38247923 10.3390/bioengineering11010045 PMC10813760

[198] Guerriero F, Ricevuti G. Extremely low frequency electromagnetic fields stimulation modulates autoimmunity and immune responses: a possible immuno-modulatory therapeutic effect in neurodegenerative diseases. Neural Regen Res. 2016;11:1888–95. 28197174 10.4103/1673-5374.195277 PMC5270416

[199] Gabriel S, Lau RW, Gabriel C. The dielectric properties of biological tissues: II. Measurements in the frequency range 10 Hz to 20 GHz. Phys Med Biol. 1996;41:2251–69. 8938025 10.1088/0031-9155/41/11/002

[200] Palmqvist S, Schöll M, Strandberg O, Mattsson N, Stomrud E, Zetterberg H, Earliest accumulation of β-amyloid occurs within the default-mode network and concurrently affects brain connectivity. Nat Commun. 2017;8:1214. 29089479 10.1038/s41467-017-01150-x PMC5663717

[201] Fuller JT. Modeling Alzheimer’s Disease: a Statistical Approach to Understanding Pathogenesis Across Brain Regions [dissertation]. Spanish: Texas A&M University; 2016.

[202] Braak H, Thal DR, Ghebremedhin E, Del Tredici K. Stages of the pathologic process in Alzheimer disease: age categories from 1 to 100 years. J Neuropathol Exp Neurol. 2011;70:960–9. 22002422 10.1097/NEN.0b013e318232a379

[203] Walker LC. Aβ Plaques. Free Neuropathol. 2020;1:1–31. 10.17879/freeneuropathology-2020-3025 PMC7745791 33345256

[204] Lucey BP, Mawuenyega KG, Patterson BW, Elbert DL, Ovod V, Kasten T, Associations Between β-Amyloid Kinetics and the β-Amyloid Diurnal Pattern in the Central Nervous System. JAMA Neurol. 2017;74:207–15. 27992627 10.1001/jamaneurol.2016.4202 PMC5305432

[205] Allen RM, Scanlan JM, Gama-Chonlon L. Bilateral rTMS Shows No Advantage in Depression nor in Comorbid Depression and Anxiety: A Naturalistic Study. Psychiatr Q. 2024;95:107–20. 38127248 10.1007/s11126-023-10062-7

[206] Lai H Biological effects of radiofrequency electromagnetic field. Encycl Biomater Biomed Eng. 2005;10:1–8.

[207] Adair ER, Petersen RC. Biological effects of radiofrequency/microwave radiation. IEEE Trans Microw Theory Tech. 2002;3:953–62.

[208] Perez F, Millholland G, Peddinti SV, Thella AK, Rizkalla J, Salama P, Electromagnetic and Thermal Simulations of Human Neurons for SAR Applications. J Biomed Sci Eng. 2016;9:437–44. 27617054 10.4236/jbise.2016.99039 PMC5014390

[209] Perez FP, Arvidson DM, Taylor TP, Rahmani M, Rizkalla M. Numerical Analysis and Design of an EMF Birdcage Wearable Device for the Treatment of Alzheimer’s Disease: A Feasibility Study. J Biomed Sci Eng. 2022;15:219–27. 36032690 10.4236/jbise.2022.158020 PMC9406889

[210] Perez FP, Bandeira JP, Morisaki JJ, Krishna Peddinti SV, Salama P, Rizkalla J, Antenna Design and SAR Analysis on Human Head Phantom Simulation for Future Clinical Applications. J Biomed Sci Eng. 2017;10:421–30. 28959376 10.4236/jbise.2017.109032 PMC5613941

[211] Perez FP, Rahmani M, Morisaki J, Amran F, Bakri S, Halim A, Numerical Modeling and Computer Simulation of a Meander Line Antenna for Alzheimer’s Disease Treatment, a Feasibility Study. J Biosci Med (Irvine). 2023;11:177–85. 36945328 10.4236/jbm.2023.112013 PMC10026125

[212] Durney CH, Massoudi H, Islanker MF. Radiofrequency radiation dosimetry handbook. 4th ed. USAF School of Aerospace Medicine, Aerospace Medical Division (AFSC), Brooks Air Force Base; 1986.

[213] Winter L, Özerdem C, Hoffmann W, Santoro D, Müller A, Waiczies H, Design and evaluation of a hybrid radiofrequency applicator for magnetic resonance imaging and RF induced hyperthermia: electromagnetic field simulations up to 14.0 Tesla and proof-of-concept at 7.0 Tesla. PLoS One. 2013; 8:e61661. 23613896 10.1371/journal.pone.0061661 PMC3632575

[214] Andrade-Guerrero J, Santiago-Balmaseda A, Jeronimo-Aguilar P, Vargas-Rodríguez I, Cadena-Suárez AR, Sánchez-Garibay C, Alzheimer’s Disease: An Updated Overview of Its Genetics. Int J Mol Sci. 2023;24:3754. 36835161 10.3390/ijms24043754 PMC9966419

[215] López-Otín C, Blasco MA, Partridge L, Serrano M, Kroemer G. Hallmarks of aging: An expanding universe. Cell. 2023;186:243–78. 36599349 10.1016/j.cell.2022.11.001

[216] Llanos-González E, Henares-Chavarino ÁA, Pedrero-Prieto CM, García-Carpintero S, Frontiñán-Rubio J, Sancho-Bielsa FJ, Interplay Between Mitochondrial Oxidative Disorders and Proteostasis in Alzheimer’s Disease. Front Neurosci. 2020;13:1444. 32063825 10.3389/fnins.2019.01444 PMC7000623

[217] Ben-Zvi A, Miller EA, Morimoto RI. Collapse of proteostasis represents an early molecular event in Caenorhabditis elegans aging. Proc Natl Acad Sci U S A. 2009;106:14914–9. 19706382 10.1073/pnas.0902882106 PMC2736453

[218] Cozachenco D, Ribeiro FC, Ferreira ST. Defective proteostasis in Alzheimer’s disease. Ageing Res Rev. 2023;85:101862. 36693451 10.1016/j.arr.2023.101862

[219] Dai C, Sampson SB. HSF1: Guardian of Proteostasis in Cancer. Trends Cell Biol. 2016;26:17–28. 26597576 10.1016/j.tcb.2015.10.011 PMC4722819

[220] Wang B, Liu S, Hao K, Wang Y, Li Z, Lou Y, HDAC6 modulates the cognitive behavioral function and hippocampal tissue pathological changes of APP/PS1 transgenic mice through HSP90-HSF1 pathway. Exp Brain Res. 2024;242:1983–98. 38935089 10.1007/s00221-024-06858-z

[221] Pasi F, Fassina L, Mognaschi ME, Lupo G, Corbella F, Nano R, Pulsed Electromagnetic Field with Temozolomide Can Elicit an Epigenetic Pro-apoptotic Effect on Glioblastoma T98G Cells. Anticancer Res. 2016;36:5821–26. 27793904 10.21873/anticanres.11166

[222] Pena-Philippides JC, Yang Y, Bragina O, Hagberg S, Nemoto E, Roitbak T. Effect of pulsed electromagnetic field (PEMF) on infarct size and inflammation after cerebral ischemia in mice. Transl Stroke Res. 2014;5:491–500. 24549571 10.1007/s12975-014-0334-1 PMC12495692

[223] Liu L, Huang B, Lu Y, Zhao Y, Tang X, Shi Y. Interactions between electromagnetic radiation and biological systems. iScience. 2024;27:109201. 38433903 10.1016/j.isci.2024.109201 PMC10906530

[224] Gruber B, Froeling M, Leiner T, Klomp DWJ. RF coils: A practical guide for nonphysicists. J Magn Reson Imaging. 2018;48:590–604. 29897651 10.1002/jmri.26187 PMC6175221

[225] Ahmad SF, Kim YC, Choi IC, Kim HD. Recent Progress in Birdcage RF Coil Technology for MRI System. Diagnostics (Basel). 2020;10:1017. 33261167 10.3390/diagnostics10121017 PMC7759766

[226] Leroy Y, Bocquet B, Mamouni A. Non-invasive microwave radiometry thermometry. Physiol Meas. 1998;19:127–48. 9626678 10.1088/0967-3334/19/2/001

[227] Gabriel C Compilation of the Dielectric Properties of Body Tissues at RF and Microwave Frequencies [Internet]. [cited 2025 Feb 1]. Available from: http://niremf.ifac.cnr.it/docs/DIELECTRIC/Report.html

[228] Braak H, Del Tredici K. The preclinical phase of the pathological process underlying sporadic Alzheimer’s disease. Brain. 2015;138:2814–33. 26283673 10.1093/brain/awv236

[229] anagopoulos DJ, Johansson O, Carlo GL. Polarization: A Key Difference between Man-made and Natural Electromagnetic Fields, in regard to Biological Activity. Sci Rep. 2015;5:14914. 26456585 10.1038/srep14914 PMC4601073

[230] Roston D, Cheatum CM, Kohen A. Hydrogen donor-acceptor fluctuations from kinetic isotope effects: a phenomenological model. Biochemistry. 2012;51:6860–70. 22857146 10.1021/bi300613e PMC3448806

